# A Basic Review of the Preliminary Evidence That COVID-19 Risk and Severity Is Increased in Vitamin D Deficiency

**DOI:** 10.3389/fpubh.2020.00513

**Published:** 2020-09-10

**Authors:** Linda L. Benskin

**Affiliations:** ^1^Independent Researcher for Improving Health in Rural Areas of Tropical Developing Countries, Austin, TX, United States; ^2^Ferris Mfg. Corp., Makers of PolyMem® Multifunctional Dressings, Ft. Worth, TX, United States

**Keywords:** vitamin D, COVID-19, health disparities, minority health, vitamin D deficiency, preventive medicine

## Abstract

As the world's attention has been riveted upon the growing COVID-19 pandemic, many researchers have written brief reports supporting the hypothesis that vitamin D deficiency is related to the incidence and severity of COVID-19. The clear common thread among the top risk groups—vitamin D deficiency—may be being overlooked because of previous overstated claims of vitamin D benefits. However, the need to decrease COVID-19 fatalities among high-risk populations is urgent. Early researchers reported three striking patterns. Firstly, the innate immune system is impaired by vitamin D deficiency, which would predispose sufferers to viral infections such as COVID-19. Vitamin D deficiency also increases the activity of the X-chromosome-linked “Renin-Angiotensin” System, making vitamin D deficient individuals (especially men) more susceptible to COVID-19's deadly “cytokine storm” (dramatic immune system overreaction). Secondly, the groups who are at highest risk for severe COVID-19 match those who are at highest risk for severe vitamin D deficiency. This includes the elderly, men, ethnic groups whose skin is naturally rich in melanin (if living outside the tropics), those who avoid sun exposure for cultural and health reasons, those who live in institutions, the obese, and/or those who suffer with hypertension, cardiovascular disease, or diabetes. And thirdly, the pattern of geographical spread of COVID-19 reflects higher population vitamin D deficiency. Both within the USA and throughout the world, COVID-19 fatality rates parallel vitamin D deficiency rates. A literature search was performed on PubMed, Google Scholar, and RSMLDS, with targeted Google searches providing additional sources. Although randomized controlled trial results may be available eventually, the correlational and causal study evidence supporting a link between vitamin D deficiency and COVID-19 risks is already so strong that it supports action. The 141 author groups writing primarily about biological plausibility detailed how vitamin D deficiency can explain every risk factor and every complication of COVID-19, but agreed that other factors are undoubtedly at work. COVID-19 was compared with dengue fever, for which oral vitamin D supplements of 4,000 IU for 10 days were significantly more effective than 1,000 IU in reducing virus replication and controlling the “cytokine storm” (dramatic immune system over-reaction) responsible for fatalities. Among the 47 original research studies summarized here, chart reviews found that serum vitamin D levels predicted COVID-19 mortality rates (16 studies) and linearly predicted COVID-19 illness severity (8 studies). Two causal modeling studies and several analyses of variance strongly supported the hypothesis that vitamin D deficiency is a causal, rather than a bystander, factor in COVID-19 outcomes. Three of the four studies whose findings opposed the hypothesis relied upon disproven assumptions. The literature review also found that prophylactically correcting possible vitamin D deficiency during the COVID-19 pandemic is extremely safe. Widely recommending 2,000 IU of vitamin D daily for all populations with limited ability to manufacture vitamin D from the sun has virtually no potential for harm and is reasonably likely to save many lives.

**Graphical Abstract:**
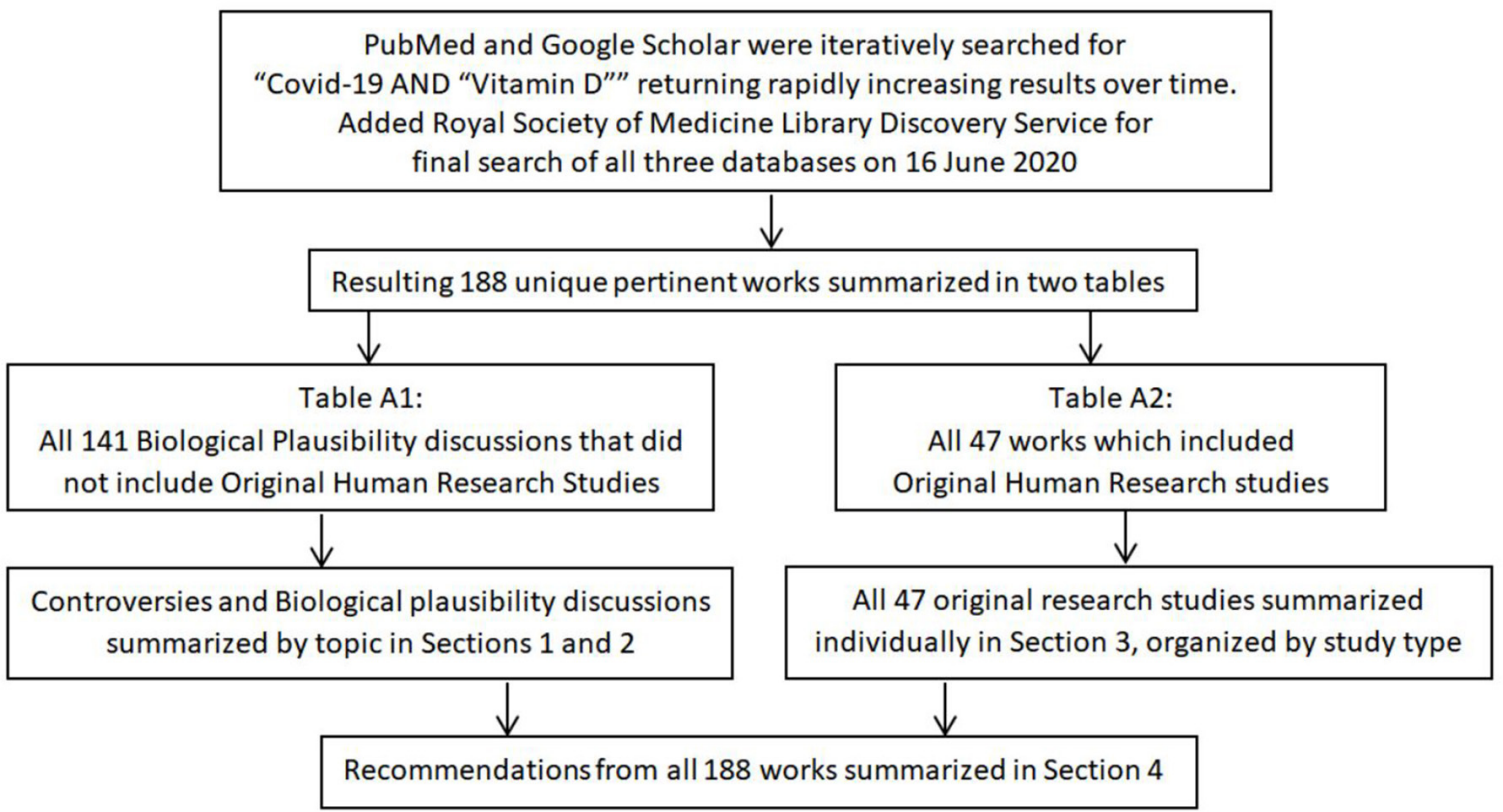
Search and reporting method.

## Introduction

COVID-19 was first recognized in December of 2019 (1, 2^p^)^1^. By January of 2020 it was clear the elderly are by far the most likely succumb to COVID-19 pneumonia, which is caused by a “cytokine storm.” (6, 7). Later, male sex, obesity, and possessing naturally melanin-rich skin while living outside of the tropics came to be known as the highest risk factors after older age (2^p^, 8–13, 14^p^, 15, 16). Unlike influenza, children under age 10 are almost completely spared in COVID-19 (17, 18). This unusual risk factor pattern presented a mystery that spawned studies showing that COVID-19 fatalities are especially high in areas with lower levels of sunshine due to latitude or air pollution, except when population vitamin D intake is high (9, 10, 12, 19–24). In fact, the risk groups for severe COVID-19 match the risk groups for vitamin D deficiency exactly, and there is biological plausibility: vitamin D is known to modulate the immune system, helping prevent both an under-reaction that allows upper respiratory infections to be contracted, and the over-reaction referred to in COVID-19 as the “cytokine storm” (see section Biological Plausibility Discussions) (19, 25, 26). This review explores the evidence related to the hypothesis that vitamin D deficiency increases both COVID-19 rates and illness severity.

### The Vitamin D Debate

The discussion of idea that the top risk groups for severe COVID-19 complications tend to have vitamin D deficiency (Table 1) was initially popularized, not by the scientific community or governmental bodies, but rather, by some entertainers and bloggers, who recommended supplements to their audiences (40–42). This led some in the scientific community to respond with either agreement or disapproval. Trinity College Dublin researchers quickly issued a news release urging the Irish government to change their recommendations for vitamin D supplements in light of evidence of an association between vitamin D levels and COVID-19 mortality (43). However, most governments, medical organizations, and key opinion leaders give one or more of these four reasons not to recommend vitamin D supplements: past claims for vitamin D benefits were overstated, evidence for a link to COVID-19 is insufficient, overdoses are theoretically possible, and the public might believe that taking vitamin D supplements will make them “immune” to COVID-19 (44–53).

**Table 1:** Classification of vitamin D levels (serum 25(OH)D levels): (3–5, 12, 27–39).

**Classification**	**Nanograms**	**Nanomoles**	**Recommended D**
Danger of toxicity	>100 ng/ml^*^	>250 nmol/l	
Normal or optimal	>30 ng/ml	>75 nmol/l	400–4,000 IU/day
Insufficient	21–29 ng/ml	51–74 nmol/l	4,000–6,000 IU/day
Deficient	11–20 ng/ml	26–50 nmol/l	7,000 IU/day
Severely deficient (often not distinguished from deficient)	<10 ng/ml	25 nmol/l	10,000 IU/day x 1 month or 500,000 IU x 1

* *some sources found that 150 ng/ml was not harmful*.

Although the International Association for Gerontology and Geriatrics (IAGG) Asia/Oceania Region COVID-19 Prevention Statement acknowledged that increasing vitamin D levels could reduce infection risks in elderly individuals whose levels are insufficient, they recommended only “getting enough sunlight in the morning” without mentioning supplements (54). Two May 2020 Centre for Evidence-Based Medicine rapid reviews concluded, without discussing any of the recent studies, that there is no evidence to support a role for vitamin D in prevention or treatment of COVID-19 or the cytokine storm (45, 55). Alarmed by the media response to a literature review suggesting a link between COVID-19 and vitamin D, two Brazilian medical associations jointly published a note stating that vitamin D supplements are only approved for bone health (56–58). The high mortality rates among minorities are providing momentum for various public health program expansions, which could diminish if vitamin D deficiency, rather than access to care and economic disparities, were found to be even a partial explanation (59–62). In addition, previous studies of dubious quality suggesting that vitamin D can “cure” various chronic illnesses and may be influencing the reluctance to recommend supplements for COVID-19 (63).

However, despite these concerns, former CDC Chief Dr. Tom Frieden recommended sunshine and up to 2,000 IU/day of vitamin D as a potential preventative for COVID-19, the British Dietetic Association recommended sunshine (or 400 IU/day for those are not able to go outside due to self-isolation), and former vitamin D skeptic Dr. JoAnn Manson's calls for daily vitamin D supplements (1,000–2,000 IU/day) during the COVID-19 pandemic—if vitamin D intake is low and going outdoors is not feasible—were published on both Medscape and WebMD (19, 64–67). Medscape published a second review of the topic by McCall, in which correcting possible vitamin D deficiency was characterized as “low hanging fruit” that has no downside (68). Mitchell's brief review (20 May 2020) in a Lancet-affiliated online journal also supported the vitamin D hypothesis (69). Authors of an early meta-analysis of nine studies found that a high percentage of COVID-19 patients are either vitamin D insufficient or deficient, and that countries with lower population vitamin D status have somewhat higher COVID-19 mortality rates and somewhat lower COVID-19 recovery rates (70). In Qatar, vitamin D for prevention of COVID-19 is being proactively delivered to the homes of high-risk diabetics (71).

#### Irish Medical Journal Debate on Vitamin D Supplements During the COVID-19 Pandemic

The Irish Medical Journal hosted a six-article formal debate on the topic in response to three published reports, including one by the researchers managing Ireland's part of the 26-country longitudinal study on aging (TILDA), in their May 2020 issue (72–75). All three reports strongly recommended vitamin D supplements to help protect all adults in Ireland from COVID-19 while they are “cocooning” (not going outdoors) (72, 73) (details in Appendix).

#### Debate Over Reports Using “Big Data” (the UK Biobank and EPIC, see Results and Retrospective Chart Reviews That Are Neutral or Strongly Oppose the Hypothesis)

Three research teams relied on the 2006-2010 UK Biobank data for the vitamin D levels included in their analyses of the relationship between COVID-19 incidence and vitamin D status (13, 76, 77). Roy et al., challenged the assertion that vitamin D levels are stable over time (important since levels were assessed 10–14 years prior to the pandemic) noting that the cited study only included women and followed up for only 5 years (13, 78, 79). In fact, the cited study (Meng et al.) found that, rather than being stable, the mean 25(OH)D increased significantly (*p* < 0.05) over the 5 years, and that the increase was driven by significant (*p* < 0.05) increases among participants who were initially at risk for deficiency; supplement intake and overall vitamin D intake increased significantly (*p* < 0.05) (78). Etsy criticized a UK Biobank study's assumption that, despite government's recommendations to supplement, the participants failed to correct any vitamin D deficiency revealed by their participation (13, 80). In their preprint, Darling et al., cited a different study to support their assertion that vitamin D levels are stable over time (76, 81). However, the Norwegians in the cited study had far higher vitamin D levels than the UK Biobank participants at their initial evaluation, a subset increased their 25(OH)D levels significantly (*p* < 0.001) by initiating supplements, and, as in the study by Meng et al., vitamin D levels did increase significantly for the group as a whole over time (*p* < 0.01) (81). The authors of the most recent article using the UK BioBank study data did not address the issue of the use of potentially no longer accurate 25(OH)D levels in their preprint, but added a reference to the same Norwegian study as Darling et al., in their published article (77).

During the time frame of these three studies, COVID-19 testing in the UK was extremely limited (Raisi-Estrabragh et al., stated that most were tested only if hospitalized) (77, 79). Such limited testing, Roy asserted, raises the possibility that the authors of the first UK Biobank study accidentally included COVID-19 positive patients who were only moderately ill in their negative group (13, 79). It is also likely that the two UK Biobank studies authored later compared COVID-19 patients with patients who had serious illnesses such as influenza pneumonia, rather than with healthy individuals (76, 77). If vitamin D deficiency increases viral infection risk and severity as hypothesized, the patients in both arms of these studies could be expected to have higher deficiency and insufficiency rates than the general population. In fact, the research teams found high rates of vitamin D insufficiency and deficiency in both of their study groups (76, 77).

Three authors all pointed out that the UK Biobank studies failed to address the severity of the COVID-19 the patients experienced, which is critical to the question of whether or not vitamin D deficiency contributes to the potentially fatal cytokine storm (13, 76, 77, 79, 80, 82). Boucher cautioned against adjusting COVID-19 study data for obesity or dark-skinned ethnicity, providing empirical evidence that both directly lower 25(OH)D (83).

Grant and McDonnell formally responded to the first UK Biobank study, asserting that their multivariable model was over-adjusted because causal factors were treated as confounds, suggesting that the authors provide multiple analyses for transparency, including simple and complex models (13, 84). They also requested that the analysis be stratified by ethnicity, citing a previous study in which low 25(OH)D increased risk for preterm birth equally across ethnicities (84). They echoed Roy's concern that lack of a positive COVID-19 test result did not assure lack of infection in the UK at the time (84). Grant and McDonnell concluded by pointing out that few UK Biobank participants had 25(OH)D levels in the immune-protective range (>40 ng/ml), which would decrease the effect (13, 84).

In their response, rather than addressing the question of vitamin D deficiency being caused by old age, disability, obesity, etc., the authors of the first UK Biobank study stated that 25(OH)D cannot be a mediator because it is not the “cause” of old age, disability, etc. (84). They asserted that impaired health is more likely associated with reduced outdoor activity than with vitamin D status (84). They presented the non-significant results of an “intermediate” model that still included the deficiency-related variables of age, sex, ethnicity, and obesity, omitting only “health-related covariates” (e.g., BP, diabetes) as proof that inclusion of potential mediators did not influence their initial study results (84). The original study authors also reiterated their assertions that there is no statistical interaction between ethnicity and vitamin D deficiency and a positive COVID-19 test would ascertain more severe infections, and concluded by stating that 40 ng/ml is not deficient because in the adult UK population mean 25(OH)D levels are only 17.4 ng/ml for men under 65 years and 18.9 ng/ml for women under 65 years (84).

Although vitamin D levels are not drawn routinely, Fox used data from EPIC, a database with 15,000,000 patients across 26 states in the USA, in his analysis of the relationship between vitamin D status and COVID-19 infection, hospitalization, and mortality rates (85). DeFilipps commented succinctly, pointing out because vitamin D deficiency is ubiquitous, assuming patients with no 25(OH)D in their charts were vitamin D sufficient renders the study results study unreliable (86). DeFilipps recommended evaluating only the subgroup who were hospitalized for COVID-19 who had pre-existing conditions and known vitamin D deficiency to determine if there was a relationship between their level of deficiency, illness severity, complications, or length of stay (86).

### Defining Appropriate Serum Vitamin D Levels and Appropriate Supplementation Dosages

The 25(OH)D (serum vitamin D level) is the most reliable indicator of functional vitamin D status, but until recently, the test assays varied (87). However, past research study results can be compared by mathematically harmonizing them, and increasingly, labs are adopting LC/MS (D_2+_D_3_) as the standard, increasing consistency (87).

#### Controversy Concerning Risk of Overdose

**Figure 1:**
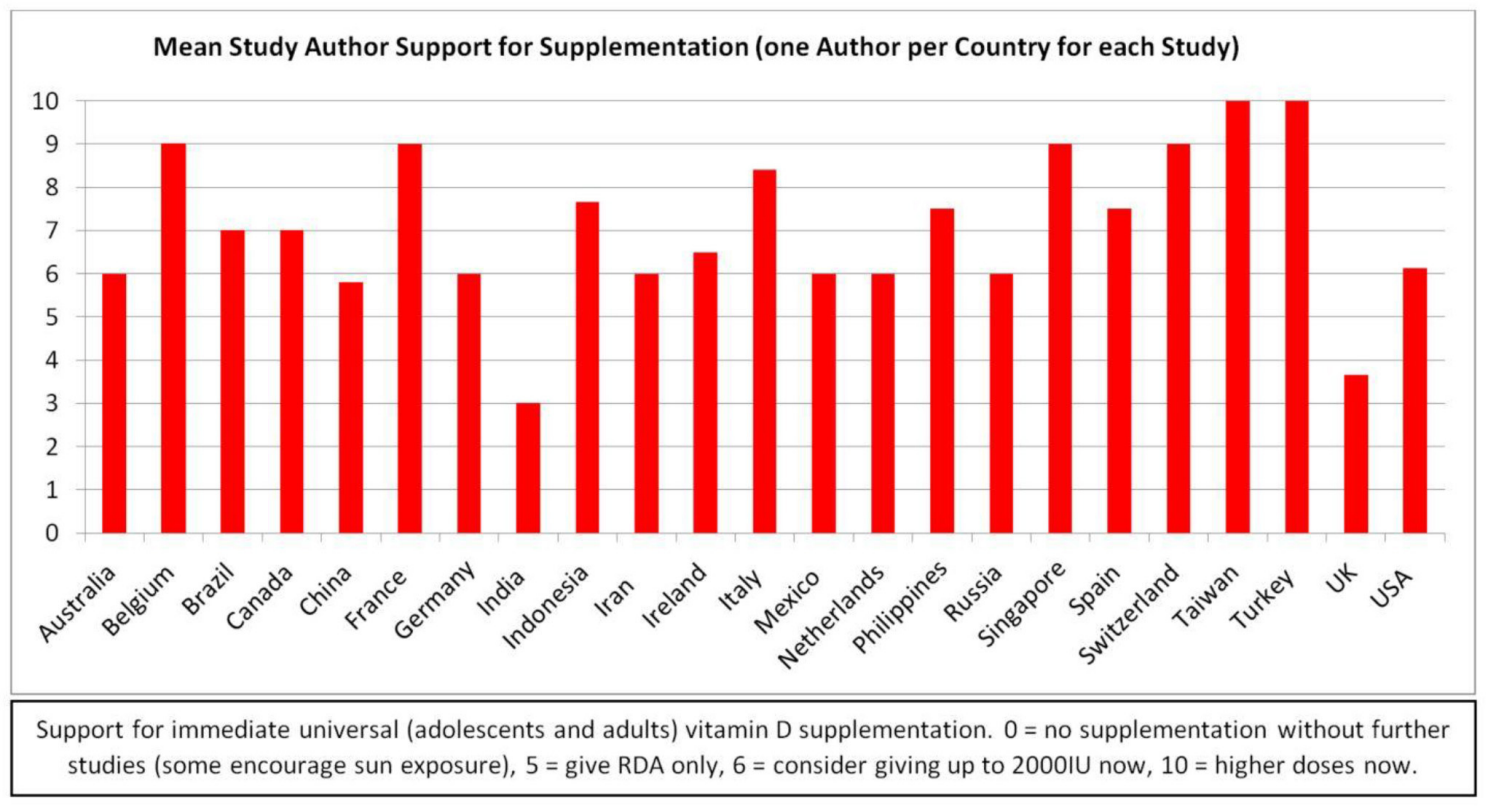
Study authors' supplementation support by country.

Food fortification was introduced shortly after the discovery of vitamin D. However, there was a dramatic increase in infants with hypercalcemia in the UK, leading to an abrupt scaling back of fortification (88). Later, a rare genetic defect, Williams–Beuren syndrome, was found to be responsible for the hypercalcemia (88). However, vitamin D toxicity concerns remain heightened, with a reluctance to recommend supplements (Figure 1). Dietary sources provide UK adults with only about 100 IUs of vitamin D per day (89, 90). During the COVID-19 pandemic, after concluding that vitamin D is likely to reduce acute respiratory tract infection risk, and that 10,000 IU/day is safe, the NHS paradoxically recommended only 400 IU/day “to protect bone and muscle health”(50, 90).

It is not considered possible to achieve toxic levels via the sun alone, and supplementation for prolonged periods brings 25(OH)D to toxic levels only if the dose is consistently extraordinarily high (40,000 IU/day for many months) (28, 88, 91, 92). The average naturally acquired 25(OH)D among equatorial tribal groups is 46 ng/ml (93). Healthy lifeguards typically have 25(OH)D levels of 100–125 ng/ml (29).

The Endocrine Society found toxicity symptoms only at levels above 150 ng/ml (93). Toxicity is related to high calcium levels; 25(OH)D levels higher than 150 ng/ml in conjunction with high calcium levels produce weakness, GI symptoms and accompanying weight loss, arrhythmias, confusion, and kidney damage (28, 88, 92). Historically, toxic levels of vitamin D (>150 ng/ml) have almost exclusively been the result of industrial errors (inaccurate doses in supplements or fortified foods), and the few cases of toxicity from extremely high doses being intentionally taken for prolonged periods of time (sometimes under the direction of a health care practitioner) were rarely severe (94, 95).

#### Controversy Over Appropriate 25(OH)D Goals

In 2014, Veugelers and Ekwaru asserted that the statistical calculations to determine recommendations for vitamin D were incorrectly interpreted, leading to a US RDA (600 IU, or 700 IU/day for those over 70) that is off by a factor of more than 10 (87, 96). Heaney et al., supported the higher level in a reply, citing a recent supplementation study which supported an RDA closer to 7,000 IU/day (97). All three groups used the goal of 20 ng/ml for musculoskeletal health (70). In contrast, the Endocrine Society, aiming to optimize immune health and other aspects of vitamin D function, recommends adults take in 1,500–2,000 IU per day to maintain a 25(OH)D level of 30 ng/ml; 30 ng/ml is the NIH target level as well (29, 87).

Controversies regarding appropriate 25(OH)D, are also informed by studies of parathyroid hormone levels (29). Parathyroid hormone levels were not reduced in participants taking 15,000 IU/day, even with 25(OH)D levels above 60 ng/dl, in a study with a goal of bringing 25(OH)D levels up to at least 40 ng/dl (93). Mean serum calcium levels were not increased from baseline (93). 25(OH)D levels of up to 120 ng/dl appeared safe, and hypercalcemia and hypercalciuria were least common in participants with the highest 25(OH)D levels (calcium was not supplemented) (93). Goal 25(OH)D levels were achieved by 70% of the participants with 6,000 IU/day for normal weight participants, but 7,000 and 8,000 IU was required for overweight and obese participants, respectively (93).

Growing research suggests that 40–60 ng/ml is needed for prevention of respiratory infections, and 50–80 ng/ml is required to favorably influence hypertension and cardiovascular disease (28). In a 2019 randomized controlled trial, subjects without deficiency [initial 25(OH)D < 25 ng/ml] who took 10,000 IU/day for 3 years were slightly less likely to suffer a serious adverse event than those taking 400 IU/day (98). Mean 25(OH)D levels in the 400 IU/day group did not increase, while 25(OH)D for the 10,000 and 4,000 IU/day groups rose and then plateaued at 58 and 53 ng/dl, respectively (98).

#### Controversy Over Recommended Supplement Doses

Recommended upper limits of vitamin D supplements in the USA were relaxed after several studies demonstrated that 4,000 IU of vitamin D daily is safe (28, 75, 91). One review showed that 10,000 IU daily seemed to be the upper limit of tolerability (75). The Endocrine Society recommends up to 10,000 IU/day, particularly for obese individuals (93, 99). However, some study participants have taken 15,000 to 40,000 IU daily for at least 6 months without apparent adverse effects (91).

The European Society for Clinical Nutrition and Metabolism recommends a one-time dose of 500,000 IU IV for ICU patients who are vitamin D deficient (25(OH)D less than 20 ng/ml), based upon evidence that this practice decreases length of stay (12, 19, 100). Giving 500,000 IU enterally over 5 days increased 25(OH)D levels and decreased ICU length of stay, but giving the entire 500,000 IU in one bolus enterally did not improve 90-day mortality rates (101, 102).

Grant et al. authored an early article positing a relationship between COVID-19 and vitamin D which recommended 10,000 IU/day for 1 month, followed by 5,000 IU/day, with a goal 25(OH)D of 40–60 ng/ml (19). Although some other researchers agreed, many were outraged (Tables A1, A2). Kow et al., questioned both the dose and the goal, citing a robust study in which supplements decreased the incidence of acute respiratory tract infections only when 25(OH)D levels were less than 10 ng/ml, and 800 IU/day was sufficient (103, 104). Grant et al., replied with several additional studies to support their recommendation of 40–60 ng/ml as a goal, but included an example of significantly decreased incidence of respiratory infections with lesser vitamin D_3_ doses (although 800 IU was inferior to 2,000 IU, it still provided significant benefits over the placebo) (105, 106).

Sharma et al., reviewed the literature informing decisions about COVID-19 and vitamin D_3_, finding compelling evidence for 10,000 IU/day for a month, followed by 5,000 IU/day to bring 25(OH)D levels up to the target of 40–60 ng/ml, then recommended a more modest 1000–2,000 IU/day (107). One group, Quesada-Gomez et al., posited that vitamin D supplementation should be with oral calcifediol (108). However, the majority of researchers and commenters recommend vitamin D_3_ supplements of 1,000 IU−4,000 IU during “COVID-19 times,” with a goal of achieving 25(OH)D levels of 30 ng/ml (see citations for Table 1 and section COVID-19-Specific Recommendations of Experts) (109).

A meta-analysis of vitamin D supplementation to prevent acute respiratory infections found that daily vitamin D supplementation was safe and provided modest protective benefits, rising to a 70% protective effect when deficiency was corrected (104, 110). However, studies also found that large bolus doses are not particularly beneficial (104). Effective study doses of vitamin D were most often in the range of 400–2,000 IU (10–50 mcg), with the higher doses being given to adults (104). A 2020 study of pregnant women also found that daily supplementation is superior to boluses, that 2,000 IU/day was sufficient to resolve deficiency over time, and that up to 5,000 IU/day is safe (111).

## Methods: Literature Search

“COVID-19” is the MeSH term for SARS-CoV-2 disease, coronavirus 2019, COVID-19, and derivative terms. The topic of COVID-19 is a relatively new one, with the first reports published only 6 months ago (January 2020). In addition, because vitamin D supplementation is controversial, publication bias is a significant concern. Consequently, a significant percentage of the pertinent literature is found only on preprint services, most of which are captured by Google Scholar. PubMed casts a wider net than MEDLINE. Therefore, initially, PubMed and Google Scholar were searched for “COVID-19” AND “Vitamin D” (date range, 2020, omitting citations and patents, no language limitations). Repeated searches confirmed the growing interest in the hypothesis that vitamin D deficiency may play an important role in the COVID-19 pandemic (3, 10, 12, 19, 25, 110, 112–127) From May 2 to May 19, Google Scholar hits increased from 49 to 88 and PubMed hits increased from 17 publications to 32. By June 16, the Google Scholar search retrieved 158 possible references and the PubMed publications on the topic had increased to 69. Using the same search terms, the author also accessed the Royal Society of Medicine Library Discovery Service, which, on 16 June, 2020, provided 144 results from academic journals, reports, magazines, and electronic resources.

**Figure 2:**
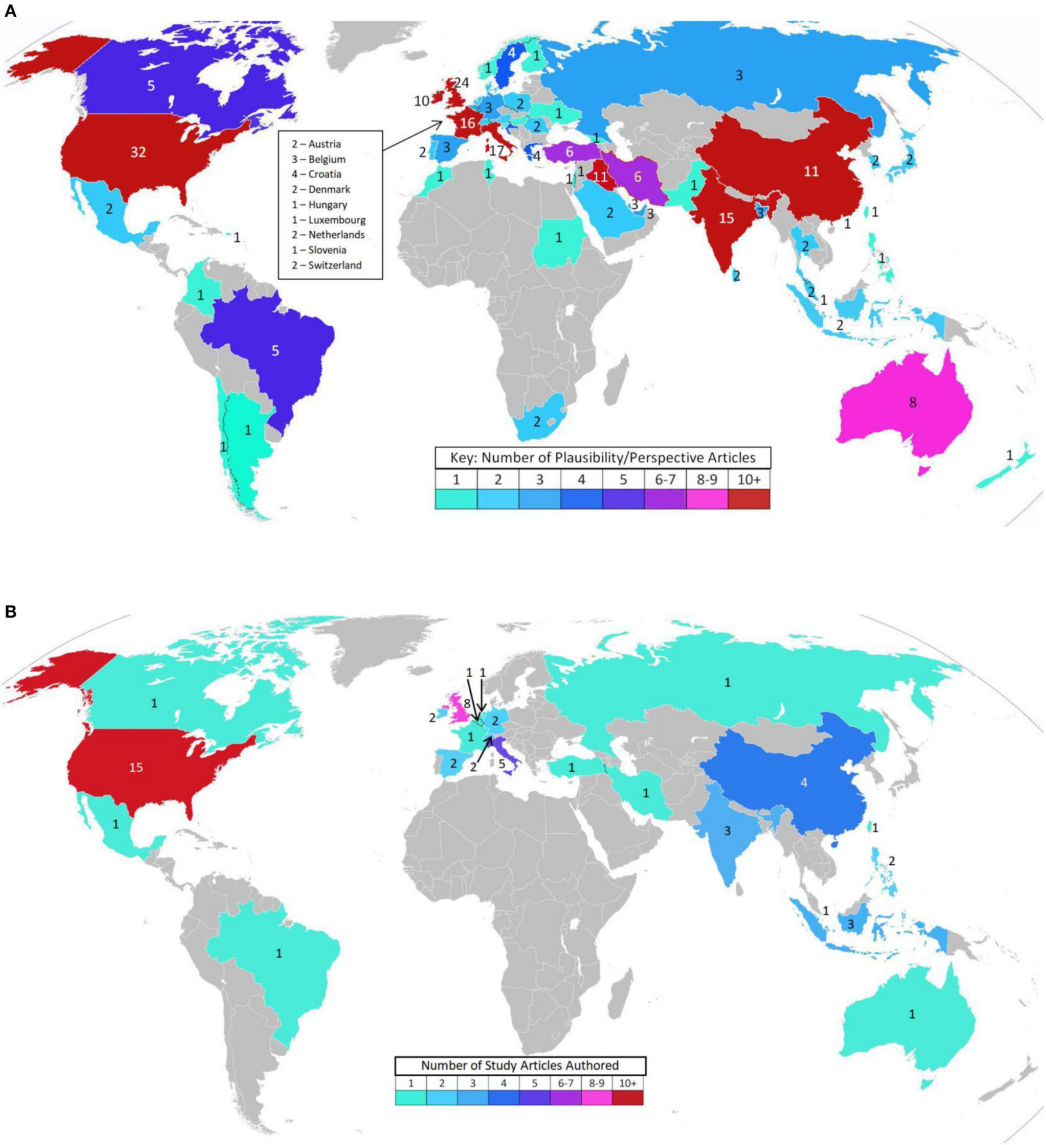
**(A)** Interest in the topic—Biological plausibility article authors' locations (141 Articles). (Blank maps from wiki images: Creative Commons, credit: Canuckguy and many others). **(B)** Interest in the topic – Original research article authors' locations (47 Articles). (Blank map from wiki images: Creative Commons, credit: Canuckguy and many others).

Duplicates were deleted and full texts obtained for every publication from all three sources as of June 16, 2020, references were scanned for additional sources, and appropriate articles found on ResearchGate and through other internet sources were added to capture how the topic is being addressed in the popular press. Authors of perspectives and studies on this topic span the globe (Figures 2A,B). Most of the research publications are quite brief, and many of the PubMed indexed articles are expert summaries of relevant data supporting the biological plausibility of the hypothesis, rather than reports of original research. Therefore, the author deemed it premature to limit this review to the “best evidence” as one would do in a formal systematic review of the literature. Rather, every publication discussing vitamin D with respect to COVID-19 found by the three formal searches as of June 16, 2020 is included in Table A1 (Biological Plausibility and In Vitro Studies, *n* = 141) or Table A2 (Original Research, *n* = 47). All original research studies (excepting *in vitro*) are summarized in the Results (section Results of Searches). However, due to space limitations, while many of the Table A1 documents are cited, few are summarized individually.

## Results of Searches

### Planned Clinical Trials

Formal clinical trials that include COVID-19 and vitamin D (including 16 on clinicaltrials.gov) include: vitamin D boluses plus other medications for COVID-19 positive patients, boluses, or moderate daily doses to prevent severe complications in at-risk populations, low-dose vitamin D as a placebo in a drug trial, genetic variant studies focused upon the interaction between vitamin D and COVID-19, and studies of vitamin D levels in patients with differing severities of COVID-19 illness (45, 128–145). As late as May 19, 2019, few studies had begun recruiting. Although most of these studies are not designed to determine if daily modest vitamin D supplementation decreases either the risk of contracting COVID-19 or its severity, Dr. Manson plans to test this hypothesis (66).

Testing the hypothesis that vitamin D deficiency prior to contracting the virus increases COVID-19 rates and severity necessitates screening participants for deficiency at enrollment. Failing to correct a deficiency being considered as a potentially significant risk factor for fatal COVID-19 complications would be unethical (146). Therefore, before and after population study designs (recommending supplements to groups known to be vitamin D deficient and observing if the groups' fatality rates decline) might be more feasible than randomized controlled trials (146).

### Biological Plausibility Discussions

Many of the reports examining the relationship between vitamin D and COVID-19 present biological plausibility arguments. These reports are summarized in both Table A1 and Table A2. The main arguments are presented concisely here, citing both COVID-19 specific and primary sources.

#### Vitamin D Enhances Resistance to Viral Illnesses

Early studies of vitamin D supplementation for acute respiratory tract infections produced conflicting results (28, 104). Most studies of vitamin D for influenza prevention were conducted on healthy populations with high baseline levels, rather than on the deficient populations who would benefit most (19, 30, 121, 125, 147). Despite this, some found that higher 25(OH)D linearly enhance the innate immune response to acute winter respiratory infections, halving the incidence and significantly reducing the duration of illness (31, 148, 149).

In 2017, 25 international researchers from 23 institutions performed a meta-analysis of individual participant data from 25 high-quality randomized controlled trials of vitamin D supplementation to prevent acute respiratory tract infections to determine why the results were inconsistent (104). They found that bolus doses were not consistently protective, even in severely vitamin D deficient populations (104). Removing bolus-dose data led to consistent findings of benefit, regardless of initial vitamin D status (104). Daily or weekly vitamin D supplementation was most beneficial for participants with baseline 25(OH)D < 10 ng/ml (severe deficiency), providing more statistically significant (*p* < 0.001) protection than the (*p* < 0.02) protection vitamin D provided less deficient participants (104). The authors found that response to vitamin D supplementation is so variable that studies should base findings on changes in 25(OH)D levels, rather than relying upon the vitamin D dose given to each participant (30, 104). They also found that vitamin D supplementation is extremely safe: even large doses did not increase risk of serious adverse events, such as renal stones (104).

Historical data provides modest support for the hypothesis that populations with high vitamin D deficiency rates allow new pandemic viral strains to propagate more freely. The only recorded time period void of new strains of pandemic influenza is 1920–1957, and vitamin D food supplementation was most prevalent during the middle of that time period: from 1930 to 1950 (63). The COVID-19 pandemic began in Wuhan during a particularly dark January: 42% darker than their average January in 13 years (2007–2020) (150).

Several studies have shown that vitamin D decreases the severity of dengue fever (151). Oral vitamin D supplements of 4,000 IU for 10 days were significantly more effective than 1,000 IU in reducing dengue virus replication and controlling the damaging cytokine hyper-reaction (151–154). Vitamin D supplementation also reduced rotavirus replication in pigs (154). A recent review article by Sharma et al., summarized biological plausibility arguments and found that vitamin D deficiency is associated with a wide range of viral illnesses, and that vitamin D supplementation was both preventative and decreased severity, limiting hyper-inflammatory complications (107).

In the lungs, formation of the peptide LL37, an innate immune system component that, among other things, attacks enveloped viruses such as SARS-CoV-2 and modulates the immune system, requires sufficient vitamin D levels (28, 32, 117, 155). LL37 is inhibited by carbon and other nanoparticles in air pollution (32). Therefore, vitamin D deficient individuals can be expected to be at increased risk of both developing COVID-19 and experiencing the “cytokine storm” if they become infected, particularly in areas of the world with high levels of air pollution (32, 117).

#### How Vitamin D May Decrease Serious COVID-19-Associated Complications

During the “Spanish flu” pandemic of 1918-1919, deaths were substantially reduced when patients were treated in “open air” hospitals with access to sunlight, perhaps due to vitamin D's “cytokine storm” suppression (63, 150, 156). In the deep south, dramatically increased incidence of pneumonia led to much higher Spanish flu case fatality rates for African Americans than for whites (19). COVID-19 usually produces mild symptoms in the seemingly-vulnerable homeless, who are disproportionately outdoors, despite the population skew toward older males and African Americans (157–159). Prior to antibiotics, cod liver oil, UVB phototherapy, and sunshine, all of which are vitamin D sources, were considered successful treatments for tuberculosis (99).

Vitamin D enhances the innate immune response while, paradoxically, protecting against excessive inflammation by suppressing TNFα and the cytokines (e.g., IL-6, IL-17) implicated in severe COVID-19, and elevating anti-inflammatory IL-10 (19, 28, 31, 33, 45, 91, 149, 160–169). Many of the articles referenced here include detailed descriptions of the role of vitamin D in preventing a “cytokine storm” and several authors, including Meftahi et al., and Biesalski, added a series of cartoons to their papers to simplify the concept (167, 168).

Given that vitamin D decreases pro-inflammatory IL-6 and that IL-6 is implicated in the COVID-19 “cytokine storm,” (170) and finding that mean IL-6 levels are higher in males and African Americans and increase with age and obesity (groups with increased risk for COVID-19 mortality), Silberstein went on to evaluate the possibility that vitamin D deficiency causes upregulation of IL-in high risk individuals prior to exposure to COVID-19, increasing their likelihood of developing fatal COVID-19 complications (171). Using detailed IL-6 data from Tuscany, Italy, Silberstein found a strong correlation between age stratified COVID-19 deaths in Italy and mean IL-6 levels [*r*(6) = 0.9837, *p* = 0.00025] (171). Data for a similarly detailed analysis for sex, obesity, and ethnicity was not available (171). The authors note that IL-6 is generally low in children, but it is high for a brief time in early childhood, which could explain the Kawasaki-like COVID-19 sequela in some children (171).

Vitamin D also helps prevent viral infections from progressing to pneumonia by tightening cell junctions (19, 28, 165, 172) And, vitamin D's influence on the coagulation pathway decreases risk of acute respiratory distress syndrome as it decreases thrombosis risks (114, 127, 157, 162, 169, 173). Therefore, correcting vitamin D deficiency might help prevent COVID-19 illness AND help limit complications when prevention is unsuccessful (25, 28, 114, 169).

Daneshkhah et al., proposed that Vitamin D deficiency causes C-reaction protein (CRP) levels to rise, thus increasing the likelihood of a cytokine storm (174). The authors found that CRP and vitamin D status are inversely related in healthy individuals (174). CRP levels were increased in severe COVID-19 patients, but because CRP is a marker for inflammation, it was unclear if this was a cause or an effect (174). The authors used population data from 10 countries to show a possible link between vitamin D status and the adaptive average case mortality ratio, and provided significant biological plausibility arguments in support of the hypothesis (174). The authors proposed further studies to determine if COVID-19 patients with high CRP are deficient in vitamin D (174).

### Risk for Severe COVID-19 Parallels Risk for Vitamin D Deficiency

Many authors, some with compelling statistical analyses, propose vitamin D deficiency from low sunlight levels (Nordic countries have high vitamin D intake) to explain the geographic distribution of severe COVID-19 (9, 10, 26, 34, 112, 122, 150). Italy and Spain have very high vitamin D deficiency rates (12, 27, 34, 63, 122, 175). First-generation non-Western immigrants, even in countries with low overall rates, are often vitamin D deficient (176–178). Vitamin D deficiency is especially common in the elderly, in part because synthesis from sunlight is muted in old age (19, 31, 34, 116, 122, 179–182). Naturally melanin-rich skin increases vitamin D deficiency risks, particularly in high latitudes (13, 34, 112, 116, 122, 148, 181). It takes significantly more sunlight exposure for someone with dark skin to attain the benefits that someone with lighter skin receives (14). Lower 25(OH)D is associated with diabetes, hypertension, cardiovascular disease, and COPD risk (19, 25, 31, 34, 183). Dialysis patients are often severely vitamin D deficient (184). Up to 50% of US nursing home patients, and 75% of institutionalized people in general, are vitamin D deficient (122, 182, 185).

The UK's low sunlight levels have been posited in the public press as an explanation for why health workers with naturally melanin-rich skin (mostly nurses and physicians) are so disproportionately represented on the Telegraph's tribute wall (186, 187). The only postpartum COVID-19 fatality in the UK was a vitamin D deficient diabetic Pakistani woman who suffered a thrombotic complication (188).

Current increased “stay at home” regulations and increased boredom and stress can be expected to result in eating patterns which increase obesity and the comorbidities with which it is associated (189). In part because vitamin D is fat-soluble, obese individuals have increased daily vitamin D intake requirements and are often deficient (25, 34, 57, 91, 175, 190, 191). In addition, vitamin D deficiency causes the body to store more fat by increasing parathyroid hormone levels (192). Obesity is a major risk factor for fatal COVID-19 complications, particularly in younger adults (34, 190). Ekiz et al., found that increasing vitamin D levels makes it easier to lose excess weight, which could lower individual COVID-19 risk (192).

Recent studies in Ireland and Switzerland both found that older males are at even higher risk of vitamin D deficiency than older females (72, 124). Vitamin D deficiency increases the X-chromosome linked “Renin-Angiotensin” System (RAS) activity, making men more susceptible to ACE2 receptor dysregulation and theoretically, to increased COVID-19 morbidity (25, 33, 123, 165, 193, 194). Although vitamin D deficiency is not universal in severe COVID-19, every deleterious symptom can be explained by RAS over-reaction, which would occur more easily in individuals without sufficient vitamin D to control the RAS (25, 126, 165, 183).

### Evidence Informing the Hypotheses That Vitamin D Deficiency Influences COVID-19

While data from randomized controlled trials is superior, the hypothesis that vitamin D deficiency is a major contributor in COVID-19 risk and severity is already supported by 20 population-data analyses, both causal inference modeling reports, four case studies/series, one prospective correlational study, one case control study, one cohort observational study, and 10 retrospective chart reviews. One population-data analysis and three retrospective chart reviews supported the dissenting view. One population-data analysis, one retrospective chart review, and the lone systematic review were neutral. Recognizing that truth is not exposed by the mere tallying of positions, but rather, by evaluating the specifics of the data and the strength of the study designs, all 47 studies are summarized here and in Table A2.

#### Analyses of Population Data

Bäcker asked whether temperature or radiance could explain the speed and level of geographic spread of COVID-19 (150). Every location with over 2000 cases by March 15, 2020 had an average temperature of 10°C or lower (150). And, over a longer time period, locations with 4-week temperature averages under 14°C when they reached 100 cases all had faster growth than any of the warmer locations (*p* = 0.0001) (150). The same analysis using deaths instead of cases yielded a similar negative correlation (*p* < 0.02) (150). However, Finland, Norway, and Russia, all reaching 2000 cases after March 15, did not conform to the pattern, leading to a study of sunlight (150). Indeed, irradiance and cloudopacity better accounted for all the of data (*p* < 0.01) (150). Bäcker suggests (with data to back up his hypothesis) that increased cloudiness and air pollution can explain why in Korea, Daegu had 10 times as many cases of COVID-19 as more internationally-connected Seoul, and in Italy, Lombardy had over 10 times as many cases as more internationally-connected Lazio (Rome) (150). Multivariate regression found that the best independent predictor of COVID-19 case (*p* < 0.001) and death (*p* < 0.001) growth rates was the average zenith (most direct sun rays) when the location reached its 100th case (150) Zenith, correlated with both irradiation (*p* < 0.01) and temperature (*p* < 0.001), explained the lower growth rate in Finland, Norway, and Russia, and fully accounted for the variance from both (150). No association with increased travel or visiting was found (150). Bäcker concluded that sunlight leads to less COVID-19 transmission, likely due to both the direct result of irradiation and increased vitamin D, and thus, advising people to stay indoors rather than opening up outdoor recreation areas during the COVID-19 pandemic appears to be a poor choice (150).

In contrast, Yao et al., found no association between COVID-19 transmission rates and temperature or UV radiation across the 62 cities (of 224) in China with at least 50 cases at the peak of the outbreak (10 Feb) and at least 10 cases remaining on 9 March (195). However, the authors note that their study examined data from early January to early March, 2020, a time during which strict travel restrictions were put into place to prevent COVID-19 transmission in China (195, 196). It is possible that many of the cities in which UV light or temperature effectively reduced transmission were eliminated from the study because they no longer had the minimum of 10 cases by 9 March.

Two statistical analyses of geographical areas in the USA addressed the question of whether high COVID-19 fatality rates in African Americans could be explained by income levels (14, 197). Bäcker's initial statistical analysis from 8 cities and states that provide a racial breakdown of COVID-19 victims found that race-based fatality rate differences diminish in direct proportion to available sunlight, with COVID-19 deaths among blacks in Detroit at 193% higher than the percent-black area population, but only 7% higher in Florida (Pearson −0.76, *p* < 0.05) (14). African Americans comprise 26% of Milwaukee County's population, half of their COVID-19 cases, and 81% of its deaths (14). This led to an exploration of the hypothesis that rather than socioeconomics (lower incomes, jobs that do not permit social distancing) being solely responsible, irradiance may play a large role in the disproportionate COVID-19 morbidity and mortality rates among African Americans in the USA (14). In Michigan, the state with the highest racial disparity in COVID-19 deaths, a county-by-county analysis showed that percent African American, but not percent over 65 years, median income, median age, or number of people per household, significantly (*p* < 0.05) correlated with COVID-19 morbidity (14).

Similarly, Li et al., focused on US counties with at least 50 COVID-19 cases (661 counties) and those with at least 10 deaths (221 counties), grouping them into quartiles and comparing highest to lowest (197). Multivariate analysis demonstrated that “percent black” predicted county cases and fatalities, even after controlling for other demographics, socioeconomics, and comorbidities (197). Higher daily temperatures decreased county case numbers, but not mortality rate (197). They proposed vitamin D deficiency among black Americans as a “unifying theory” to explain their results (197).

Laird et al., plotted COVID-19 mortality/million against mean 25(OH)D levels for twelve European countries, finding a significant correlation (*p* = 0.046) (27). Panarese and Shahini ranked the 108 countries with at least 100 COVID-19 cases on 2 April 2020 by latitude, demonstrating visually that, overall, deaths per million were higher in the northern-most countries, whose citizens would be the most likely to be vitamin D deficient from the dark winter (198). Following up on Panarese and Shahini's work, Rhodes et al., compared the 120 countries with more than 150 COVID-19 cases by 15 April 2020, finding that COVID-19 mortality rates were significantly correlated with latitude (*r* = 0.53, *p* < 0.0001) (26). Rhodes et al., used a simple scatter-graph to illustrate that the COVID-19 mortality rates per million population were dramatically lower in countries with capitals south of 35°N, where sunshine in the time immediately preceding the pandemic made maintaining vitamin D levels possible (26).

Ilie et al., reported a significant correlation between low mean vitamin D levels across 20 European countries and both COVID-19 fatalities/million population (*p* = 0.05) and COVID-19 cases/million population (*p* = 0.050) (122). Kumar and Srivastava objected to Ilie et al.,'s study, stating that the correlation was being stretched by the media to claim that vitamin D supplements may reduce COVID-19 mortality rates by 50% (199). Expressing concern that this exaggerated claim would lead to fatal overdoses, the authors conducted a statistical analysis of COVID-19 case and death rates and life expectancy using the data from Ilie et al. (122, 199). The authors asserted that because vitamin D deficiency increases with age, controlling for life expectancy would reveal the true relationship between vitamin D and COVID-19 infection and fatality rates (199). The researchers found that life expectancy was a better predictor of both COVID-19 mortality and case rates than vitamin D (199). Kumar and Srivastava did, however, call for clinical trials of vitamin D supplementation (199).

Citing Ilie et al., Singh et al. compared mean 25(OH)D levels and COVID-19 cases and deaths per million population for 20 European countries on 8 April and again on 12 May (200). The significance of the inverse correlation between vitamin D and case rates increased from *r*_(20)_: −0.4435; *R*^2^ = 0.1967 (*p* = 0.0501) in April to *r*_(20)_: −0.5504; *R*^2^ = 0.3029 (*p* = 0.0119) in May (192^p^). However, the inverse correlation for death rates decreased from *r*_(20)_: −0.4378; *R*^2^ = 0.1917 (*p* = 0.0535) to *r*_(20)_: −0.3935; *R*^2^ = 0.1549 (*p* = 0.0860) (200). Singh et al., did not discuss the possibility that vitamin D levels increased between April and May as sunshine increased, potentially protecting patients from the “cytokine storm” (200).

Notari and Torrieri's much larger, more detailed, comprehensive 126 country data review found that most of the 24 identified potential risk factors for COVID-19 propagation, including blood type, life expectancy, and even greeting habits, were significantly correlated with one another (201). Prevalence of Type-I diabetes, BCG vaccination, and vitamin D levels were the only “almost independent factors” (201). In the 42-country subset with vitamin D data and high GDP, lower mean annual levels of vitamin D were linearly related to increased COVID-19 risk (*p* = 0.006), with seasonal values (March) demonstrating even more significance (*p* = 0.002) (201).

Kara et al., mapped the population prevalence of vitamin D deficiency (< 20 ng/ml) and severe deficiency (< 10 ng/ml) against COVID-19 total fatalities for the 40 most affected countries, worldwide, finding a clear relationship (34). Regression analyses demonstrated a quadratic relationship between prevalence of vitamin D deficiency and insufficiency and COVID-19 cases (34). A histogram with regression lines illustrated the relationship between latitude, population vitamin D status, and country rank (by number of cases) (34). Finding vitamin D deficiency and COVID-19 to be related pandemics, they agreed with Grant et al., in recommending vitamin D (without high calcium) supplementation, as well as encouraging fortified food intake and increased sun (UVB) exposure (34).

Braiman noted that as of March 22, 2020, although 10% of the COVID-19 cases lived south of the Tropic of Cancer, they represented only 1% of the fatalities (146). The three exceptions could all be explained by mean population vitamin D levels (146). Nordic countries have vitamin D deficiency rates below 1% (due to diet or supplementation) and impressively low COVID-19 fatality rates, except Sweden (146, 202). In Stockholm, severe vitamin D deficiency is common among displaced Somalis, who with less than 1% of the population have suffered 40% of the COVID-19 fatalities (10, 178, 203). Indonesia straddles the equator, but its predominately Muslim women have vitamin D levels that are only half that of notoriously low Italy (146). Sunscreen use is popular in the Philippines, which may account for the high levels of vitamin D deficiency there (146). Braiman recommended ethical testing of the hypothesis that vitamin D and COVID-19 outcomes are related by encouraging supplementation in deficient populations and evaluating death rate changes (146).

Although Latinos and African Americans were found to be at higher risk of COVID-19 mortality in New York City, it is difficult to determine the influence of Latino ethnicity vs. race (over 75% of Latinos identify as non-white), because New York City does not provide sufficiently detailed data (204). In contrast, Georgia does break down COVID-19 data by both ethnicity and race (204). Black Latino COVID-19 morbidity was 123% higher than white Latino morbidity (*p* < 0.001), supporting researcher Bäcker's hypothesis that a darker complexion decreases sun exposure benefits (204). COVID-19 morbidity is 37% higher for white non-Latinos than for white Latinos (*p* < 0.0001), 689% higher for Native American non-Latinos than for their Latino counterparts (*p* < 0.01), and there were no cases of COVID-19 among Latino Asians (204). Latinos spend more time outdoors than any other racial group (85% more than African Americans) which could explain why Latinos defied externally-imposed racial disparity explanations (204). The author concluded that irradiance exposure seems to help prevent COVID-19 (204).

Countries with higher rates of vitamin D-rich sea fish consumption or food supplementation have lower COVID-19 mortality rates than adjacent countries (182). The elderly, especially in nursing homes, where 84–93% of residents in the US are vitamin D insufficient, are at highest risk for severe COVID-19 (182). Bäcker and Mageswaran evaluated vitamin D deficiency rates among elderly females and COVID-19 deaths prior to May 31st in 32 countries, finding that case fatality rates were up to twice as high in countries with high vitamin D deficiency rates (*p* < 0.04) (182). They also found that case fatality rates were significantly higher (*p* < 0.026) in countries with a high percentage of black inhabitants (182). Noting many biological plausibility arguments and vitamin D deficiency and insufficiency race disparities, the authors recommend COVID-19 prevention and treatment studies (182).

Li et al., used machine learning to produce logistic models to predict case rates, death rates, and case fatality rates in all 50 US states and 154 countries listed on the Johns Hopkins COVID-19 dashboard on 15 May 2020, assessing the interdependence of the 57 factors LASSO identified as potentially influencing COVID-19 outcomes (205). Among their many findings, Li et al., determined that higher population vitamin D intake is an independent factor in reduced COVID-19 cases (205).

Kohlmeier performed a Mendelian randomization to test the effect of latitude (a proxy for vitamin D) on rates of African American COVID-19 deaths in the 22 reporting states with more than 15 African American deaths as of 16 April 2020, finding a strong relationship (*r* = 0.427) (206). A correlational analysis found that excess mortality rates were significantly higher (*r* = 0.435, *p* = 0.02) in states with higher latitudes (206). The highest excess mortality rates were all in states near or above 40° N, where UVB intensity in winter and spring is too low to provide vitamin D (206). The African American fatality over-representation was 5.6-fold in Wisconsin compared with 1.3-fold in Florida (206).

Adding the proposed relationship between latitude and COVID-19 to knowledge that ozone filters the ultraviolet-B radiation the body requires to produce vitamin D, Alipio evaluated data from all 34 countries with April 2019 ozone data available on an open-access database (207). Kendall rank correlation test found that ozone concentration significantly (*P* < 0.001) positively correlated with COVID-19 cases, but latitude and COVID-19 cases appeared to have no relationship (207).

Recognizing the advantages of comparing cities within a single country with varied UV radiation, altitude, and weather patterns, such as consistent policies, culture, and genetic factors, Skutsch et al., conducted a multiple regression analysis of data from 45 cities in Mexico, comparing the rate of increase in cumulative COVID-19 cases and fatalities (208). Data from January was included because, while UV light's sterilization effect would be immediate, physiologic vitamin D formation precedes its impact on infection and mortality rates (208). Skutsch et al., found a negative relationship between rate of transmission and altitude (*r* = −0.354, *p* = 0.014), but temperature, relative humidity, and latitude were insignificant (208). UV levels in January correlated a bit more strongly with transmission rates (*r* = −0.369, *p* = 0.014) than UV levels during the transmission period (*r* = −0.32, *p* = 0.032), supporting the hypothesis that the influence of UV is due to vitamin D rather than sterilization (208). In contrast, UV was only marginally associated with rates of mortalities (208). Mexico City's air pollution may have explained this (208). Surprisingly, altitude and UV levels were not significantly interrelated, but their combined effect accounted for 18% of transmission rate variation (*p* = 0.0062) (208). Data for 834 individuals scattered across 561 municipalities showed that lower altitude is a highly significant (*r* = −0.35, *p* = 0.0005) predictor of vitamin D levels, perhaps influenced by the high levels of UV light in coastal cities and the cooler climate of higher altitude cities leading to more clothing coverage (208).

Noting that all five US states with fatalities greater than 5,000 and four of the five states with cases over 90,000 are in latitudes above 37°N, Li, et al., used latitude as an indicator to evaluate the relationship between sunlight, vitamin D, and COVID-19 case and death rates per 100,000 population (209). Aggregate data (22 Jan−23 May 2020) showed that states in latitudes above 37°N, when compared with states at lower latitudes, had significantly higher case rates (702 vs. 255/100 K) and death rates (43 vs. 11 deaths/100 K) (*p* < 0.001) (209). The higher case rates were not attributable to higher test rates (209). The authors suggested sunlight and vitamin D as the explanation, calling for studies to evaluate the impact of vitamin D on the prevention of COVID-19 (209).

In a less detailed study, Marik et al., evaluated the case fatality rates for all 50 US States, mapping the results to illustrate that, with the exception of states with very low population densities and Louisiana, case fatality rates increased with increasing latitude (210). The cumulative summary case fatality rate for states over 40°N was significantly higher than for states below 40°N (6.0 vs. 3.5%, *p* < 0.001) (210). Attributing the differences to vitamin D's dampening of excessive inflammation, they advocated for standard vitamin D supplement doses and further studies (210).

Moozhipurath et al., obtained UVB radiation data for 108 days (through 8 May 2020) in the 152 countries with more than 20 COVID-19 cases, beginning when the country had over 20 cases, analyzing the relationship between daily UV index (UVI—a surrogate for UVB), COVID-19 deaths, and COVID-19 cases, controlling for weather variables, including ozone levels (24). UVI increase was associated with a 1.2% decrease in the daily growth rate of cumulative COVID-19 deaths (*p* < 0.01) and a 1.0% decrease in the daily growth rate of cumulative COVID-19 case fatality rates (*p* < 0.05) (24). The authors asserted that their methods led to very conservative estimates of the effect of UVB on COVID-19 deaths, and advocated for “sensible” increased exposure to sunlight, particularly for people at high risk of vitamin D deficiency (24).

A statistical analysis by Davies et al., found that COVID-19 outbreaks with large fatality rates occurred exclusively above 30°N, with a 55:1 ratio between 30 and 55°N and more southern latitudes (63). The Epidemic Severity Index was greater than 2.5 in nine of 239 locations, all above 30°N (63). Northern outliers all had higher vitamin D population levels, southern countries with the most severe outbreaks (Philippines and Brazil) have a high vitamin D deficiency prevalence, and fatality rates are doubled by naturally melanin-rich skin in the USA and UK (63). Iran, where religious full-body clothing is worn and vitamin D deficiency is common, fared far worse than Israel, whose vitamin D deficiency prevalence is relatively low (63).

#### Causal Inference Modeling Reports

**Figure 3:**
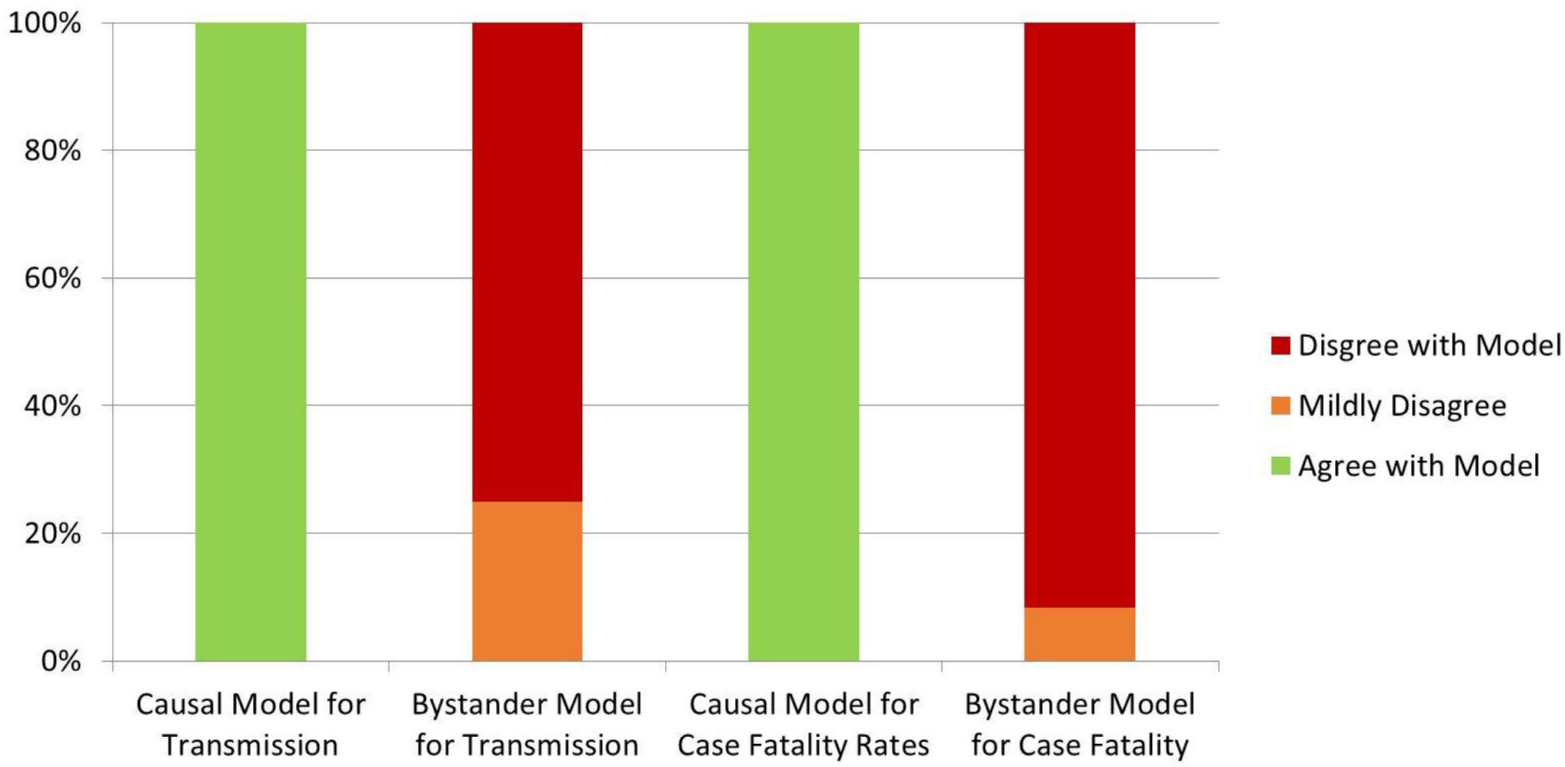
Davies et al., Causal model results (63).

Davies et al., also analyzed three potential root causes for their influence on COVID-19 outcomes, categorizing factors as lowering vitamin D, negatively influenced by low vitamin D, or vitamin D neutral (63). Environmental conditions hostile to the virus and environmental measures (e.g., distancing) decrease COVID-19 spread, but do not influence case fatality rates (63). If vitamin D is a “bystander” variable (simply a marker of bad health), case fatality rates would correlate best with vitamin D-neutral comorbidities (63). The authors provide a detailed analysis of the known COVID-19 epidemiological, latitude, and environmental data (63). A table illustrates that 16 predictions of the causal model accurately match the known facts, while 14 predictions of the bystander model strongly contradict the data and two more are not supported (Figure 3) (63).

Annweiler et al., used Hill's methodology for determining causality, which states that the more of the seven criteria are met, the stronger the claim, to evaluate the hypothesis that vitamin D is causally linked to COVID-19 outcomes (211). Vitamin D met six of the criteria, failing only on specificity (because vitamin D deficiency is high in the general population) (211). Concluding that vitamin D deficiency is highly likely to be a cause of poor COVID-19 outcomes, the authors suggest that these results, coupled with the excellent safety profile of vitamin D and lack of other treatments, support testing vitamin D as an adjuvant treatment and prophylaxis for the general population (211).

#### Case Studies and Case Series in Which Vitamin D Is Mentioned

Ahmed et al., reported that a COVID-19 positive maternity patient with diabetic ketoacidosis, vitamin D deficiency, and a history of asthma developed a fatal thrombosis 4 days post extubation (188). Horowitz et al., reported on two COVID-19 pneumonia patients with histories of immunosuppression from Lyme disease who responded to repeated doses of glutathione, along with a multitude of other drugs and remedies (212). One had a history of low vitamin D (212). Bossoni et al., reported on a 72-year-old thyroidectomized COVID-19 positive patient who experienced sudden onset severe hypocalcemia (213). Her parathyroid level was low, and she was extremely vitamin D deficient (8 ng/ml) (213). Bossoni et al., noted that home confinement can worsen vitamin D deficiency, increasing the risk of systemic infections and potentially life-threatening hypocalcemia (213).

Vitamin D deficiency is common in Indonesia, affecting 35.1% of elderly institutionalized women and 23% of the general population (214). Pinzon et al., tested 10 PCR-positive COVID-19 patients in Indonesia, finding that nine were vitamin D deficient (25(OH)D < 10 ng/ml) and the remaining patient was insufficient (25(OH)D = 20.5) (214). Finding no clinical evidence to inform the decision to provide vitamin D supplements to prevent or treat COVID-19 in their review of the literature, they called for randomized controlled trials and now prescribe all patients 2,000 IU/day (214).

#### Prospective Correlational Study, Case-Controlled Survey, and Cohort Observational Study

Vitamin D deficiency is common among Irish males (median 25(OH)D of 18.8 ng/ml for ages 40–60) (215). Faul et al., drew 25(OH)D in 33 COVID-19 positive Caucasian males over the age of 40 who were admitted to the hospital in respiratory failure without cancer, diabetes, cardiovascular disease, or chronic immunosuppressant intake in Ireland in March of 2020 (215). The 12 requiring mechanical ventilation (including all four fatalities) had mean serum 25(OH)D levels of 10.8 ng/ml, compared with 16.4 ng/ml for those requiring only oxygen (*p* = 0.03) (215). Patients with 25(OH)D < 12 ng/ml had a hazard ratio for requiring ventilator care of 3.19 (*p* = 0.03) (215). The authors concluded that low vitamin D is either a marker for poor health, or it permits pro-inflammatory changes that lead to severe COVID-19: “a thought worthy of further study” (215).

Concerned about the effects of COVID-19 on their community-dwelling Parkinson's Disease patients in Lombardy, Italy, Fasano et al., conducted telephone interviews with 1,486 patients and 1,207 family-member case controls (216). The 105 Parkinson's patients with COVID-19 and 92 family members with COVID-19 differed only in decreased shortness of breath (*p* = 0.004) and decreased hospitalization rates (*p* = 0.018) for the Parkinson's patients (216). The authors adjusted the data for the age differences between groups, thought to be due to aggressive protective measures for the elderly in the area (216). Parkinson's, hypertension, and COPD medications did not influence the likelihood of developing COVID-19, while Vitamin D supplementation was protective (*p* = 0.048).

Tan et al., compared the 26 consecutive patients 50 years or older not requiring oxygen on admission who were hospitalized immediately prior to initiation of a daily oral combination of 1,000 IU vitamin D_3_, 150 mg magnesium, and 500 mcg vitamin B_12_ with the next 17 consecutive patients meeting the same criteria to determine if these supplements altered support needs (217). Of the 9 patients supplemented within a week of symptom onset, only one required oxygen, and that was within 24 h of supplement initiation (217). Of the 8 patients supplemented over a week after symptom onset, one required ICU care within 24 h of supplement initiation, and one required oxygen 3 days later (217), Supplemented patients were less likely to need any oxygen (17.6 vs. 61.5%, *p* = 0.006) or ICU care (5.9 vs. 30.8%) (217).

#### Retrospective Chart Reviews Favoring the Hypothesis

**Figure 4:**
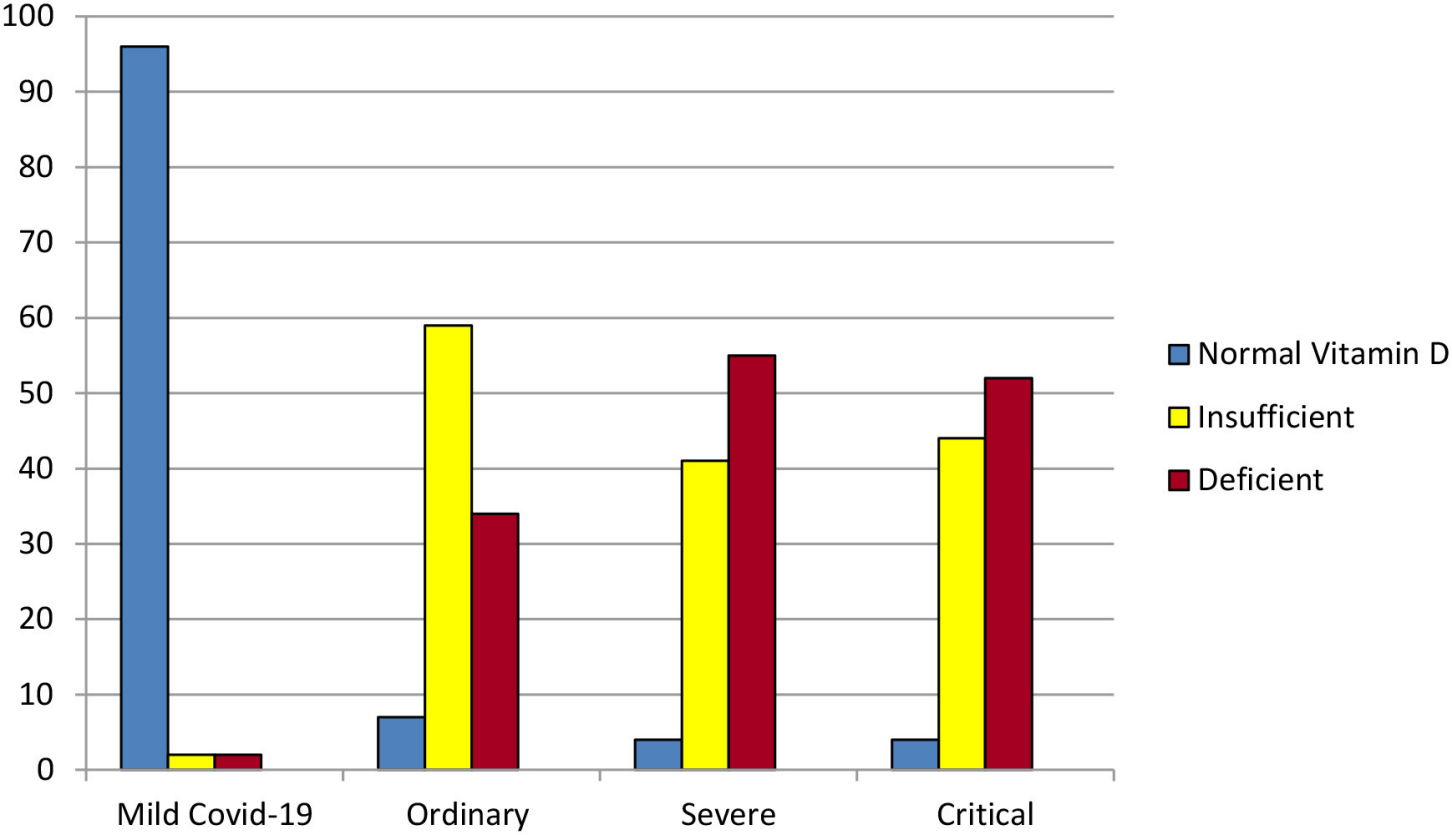
Some of the results of the retrospective chart review by Alipio et al. (3). Of the 212 hospitalized COVID-19 patients, 96% of those with mild COVID-19 had normal vitamin D levels (above 30 ng/ml). In contrast, over 50% of the patients with severe or critical COVID-19 were vitamin D deficient (level 20 ng/ml or lower).

Alipio performed a chart review using de-identified data from 212 COVID-19 patients with recorded pre-COVID-19 25(OH)D levels from three hospitals in Southern Asia in which 25(OH)D was tested initially and weekly (3). Individuals' 25(OH)D levels did not vary significantly during hospitalization, confirming that battling COVID-19 does not, in and of itself, deplete vitamin D (3). Vitamin D status (3 categories: >30, 21–29, or < 20 ng/ml) correlated significantly and linearly with more critical COVID-19 illness (4 levels clearly defined by previous researchers) (3). For each standard deviation increase in serum 25(OH)D, the odds of having a mild, rather than a critical, case of COVID-19 were almost 20 times as great (OR = 0.051, *p* < 0.001) (3) (Figure 4).

**Figure 5:**
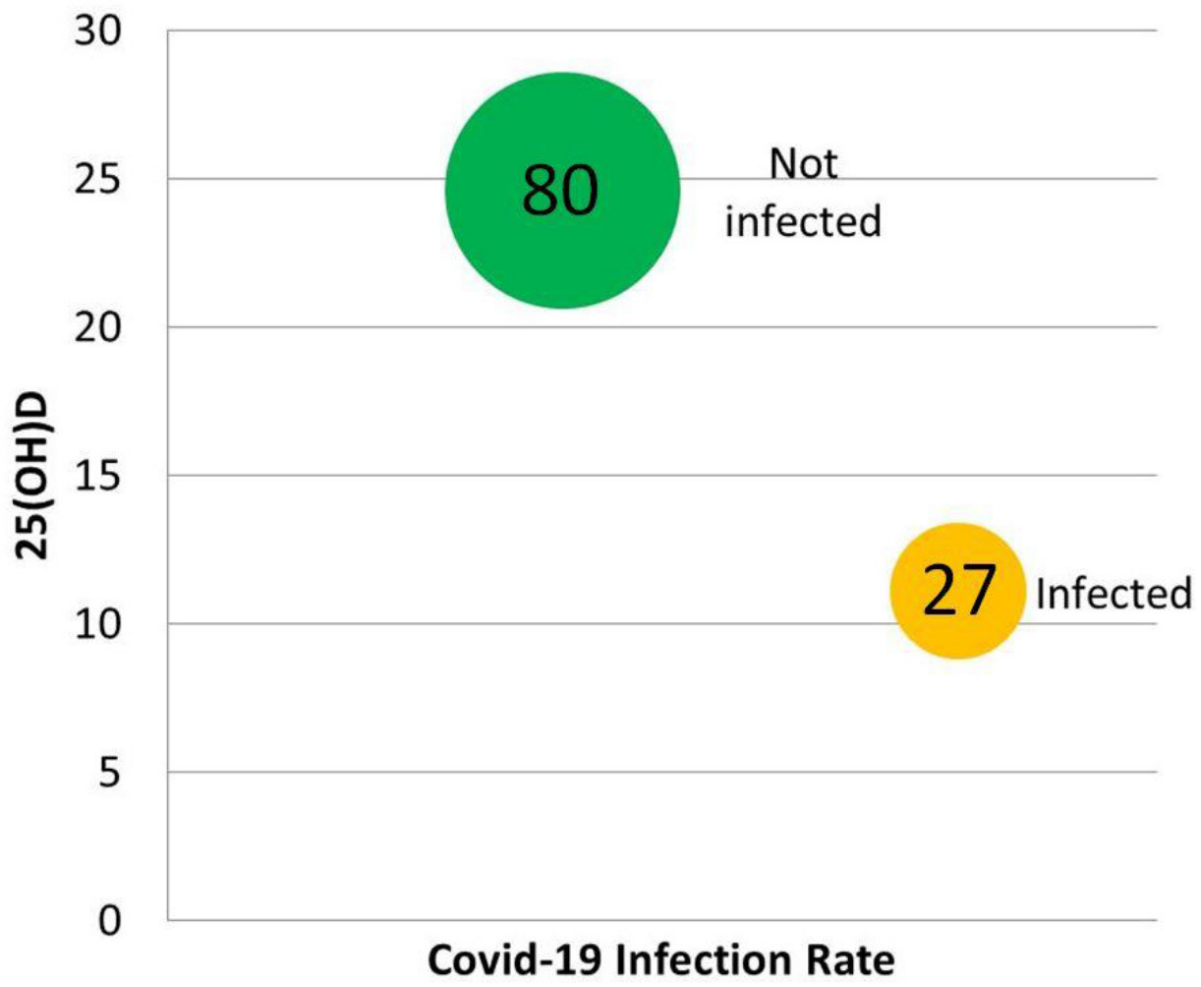
Some of the results of the retrospective chart review by D'Avolio et al. (124). In the 107 individuals tested for both 25(OH)D and COVID-19, vitamin D predicted infection.

A statistical analysis by D'Avolio et al., of records in a Swiss clinic's database for 107 symptomatic individuals obtaining a SARS-CoV-2 PCR test found that the 27 PCR-positive patients had significantly lower (*p* = 0.004) 25(OH)D (11.1 ng/ml) when compared with test-negative subjects (24.6 ng/ml) (124). PCR-positive patients were 70.4% male, while PCR-negative patients were only 48.8% male, with similar ages (124). Differences between 2019 median 25(OH)D verses PCR-positive 2020 median 25(OH)D were significant for both women (25.6 vs. 9.3 ng/ml, *p* = 0.019) and men (22.9 vs. 11.4 ng/ml, *p* = 0.005), but not for PCR-negative for either gender (Figure 5) (124).

**Figure 6:**
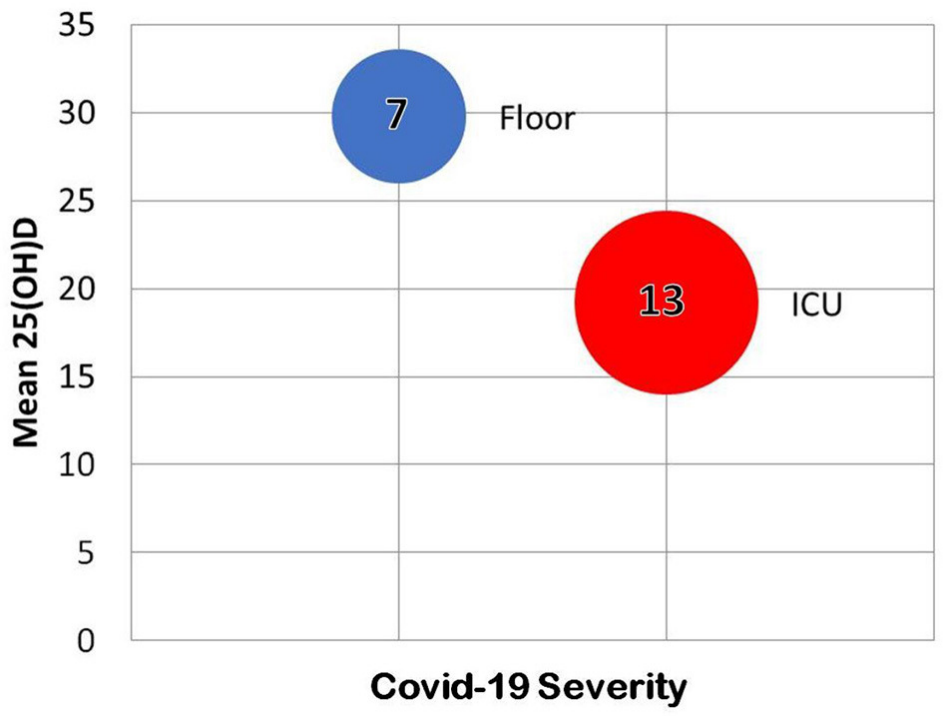
Some of the results of the retrospective chart review by Lau et al. (157). In the 20 hospitalized individuals tested for both 25(OH)D and COVID-19, vitamin D predicted severity of infection.

Lau et al., found that among all 20 COVID-19 patients with recorded 25(OH)D at a New Orleans hospital, every ICU patient under age 75 had vitamin D insufficiency (157). Eleven of 13 ICU patients had vitamin D insufficiency verses 4 of 7 patients with milder COVID-19 (157). Seven ICU patients had critically low 25(OH)D (< 20 ng/ml) and three had levels below (10 ng/ml) (157). Patients with the lowest 25(OH)D levels were African American (Figure 6) (157).

In Jakarta, Indonesia, hospitals are designed to provide patients sunlight and home patients exercise outdoors (218). In this setting, daily minutes of sunshine were compared with patient recovery, death rates, and incidence (218). Asary and Veruswati found sunshine was not related to prevention, but COVID-19 patient recovery briskness was significantly (Spearmen's α = 0.05) correlated with sunnier days (218).

Sun et al., conducted a 241 patient retrospective chart review in a hospital in Wuhan, China, using standardized definitions of mild, moderate, severe, and critical COVID-19 (219). On admission, 74.7% of patients were hypocalcemic (219). Noting that vitamin D deficiency can cause hypocalcemia, the researchers found a median 25(OH)D of 10.20 ng/ml (severe deficiency) among the 26 patients tested; none were vitamin D sufficient (219). These 26 patients had worse CRP (*p* < 0.001), D-dimer (*p* < 0.001), and parathyroid hormone (*p* = 0.048) levels (219). Calcium levels positively correlated with 25(OH)D levels (*p* = 0.004), and lower calcium levels correlated linearly with lower SpO_2_ levels (*p* < 0.001), higher complication rates (*p* < 0.001), and higher 28-day mortality rates (*p* < 0.001) (219). Vitamin D deficiency and hypoproteinemia were associated with increased mortality in critically ill patients (219).

Cuñat et al., found that although recommended for all ICU patients, vitamin D was tested in only 17 of the 226 consecutive COVID-19 patients admitted to their hospital in Spain (220). All 17 were vitamin D deficient (25(OH)D < 20 ng/ml), 13 had < 12.5 ng/ml, and three had < 5 ng/ml (220). Of these 17 patients, 35.2% had hypocalcemia and 64.7% had hypophosphatemia (220). The incidence of nosocomial infections was very high (76.5%) (220). The authors stated that vitamin D deficiency is especially problematic for COVID-19 ICU patients because vitamin D reduces pro-inflammatory and increases anti-inflammatory cytokines (220).

Raharusuna et al., conducted a retrospective chart review of 780 hospitalized test-confirmed COVID-19 patients in Indonesia (4). After controlling for age, sex, and comorbidity, both insufficient (odds ratio 7.63) and deficient vitamin D (odds ratio 10.12) were significantly associated with COVID-19 mortality (*p* < 0.001 for each) (4). Fatalities were 4.1% in patients with normal 25(OH)D, 87.8% with insufficiency, and 98.9% with deficiency (4).

**Figure 7:**
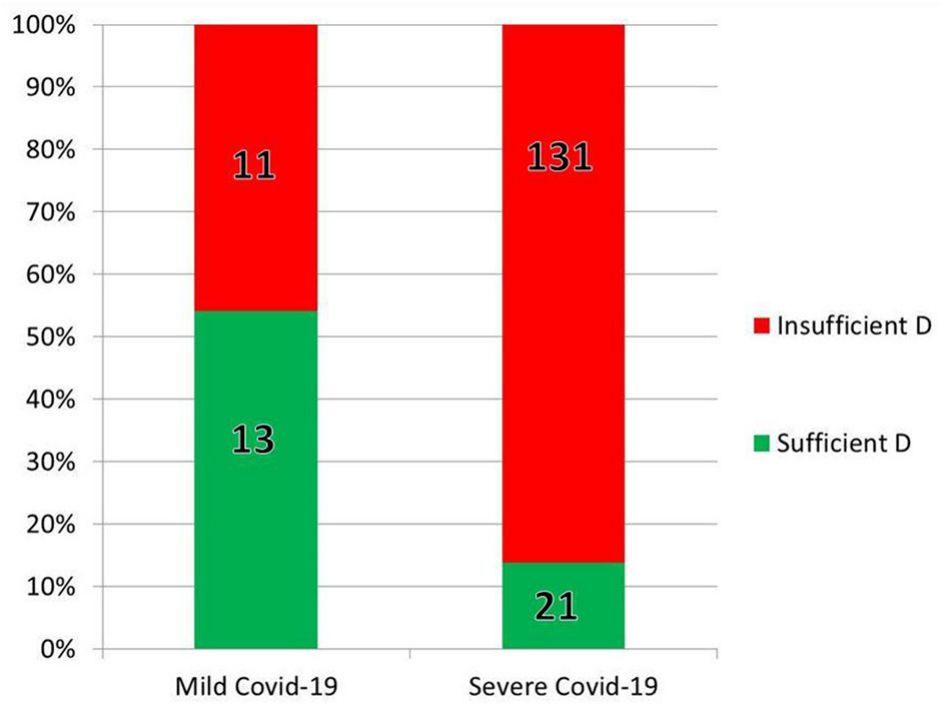
Some of the results of the retrospective chart review by Glicio et al. (5). In the 176 elderly (over age 60) hospitalized individuals tested for both 25(OH)D and COVID-19, vitamin D predicted severity of infection.

In India, Glicio et al., performed a statistical analysis on the data from the 176 COVID-19 patients 60 years or older in two tertiary medical centers whose medical records included body mass index (BMI), sex, comorbidities, clinical characteristics, and pre-hospitalization 25(OH)D (5). Over 80% were vitamin D insufficient or deficient, and of those, 72% were male (5). Inadequate 25(OH)D was strongly associated with chronic kidney disease, hypertension, and diabetes (5). Vitamin D levels were lower, with a linear distribution, in older patients (oldest age was 85) (5). Insufficient 25(OH)D was found in 45% of the 24 patients with mild COVID-19 vs. 86% of the 131 patients with severe outcomes (5). As age increased, vitamin D levels correlated linearly with outcomes, with patients over 70 suffering severe COVID-19 only if they were vitamin D insufficient (5). In contrast with obese patients, those with healthy BMIs tended to have severe COVID-19 only if they were vitamin D deficient (also a linear correlation) (Figure 7) (5).

De Smet et al., found endemic vitamin D deficiency in their area of Belgium, with lower mean levels in men than women except in summer (*p* < 0.05) (221). Children under age 18 had lower deficiency rates (*p* < 0.05) (221). Comparing 186 consecutive test-positive COVID-19 patients (109 male) with the 2,717 consecutive age-matched controls whose 25(OH)D was tested during the same season in 2019, they found that vitamin D deficiency was prevalent in controls (45.2%), but significantly (*p* < 0.05) more common in the hospitalized COVID-19 patients (58.6%) (221). The median 25(OH)D for COVID-19 patients was 18.6 ng/ml, compared with 21.5 ng/ml for controls (*p* = 0.0016) (221). Male patients were more likely than their control counterparts to be deficient (67.0 vs. 49.2%, *p* = 0.0006) (221). Vitamin D deficiency was strongly associated with more severe COVID-19 pneumonia in males (55.2% with stage 1, 66.7% with stage 2, and 74% with stage 3, *p* = 0.001), but not in females (221). Vitamin D was stable across all stages of COVID-19 for females, suggesting that the illness itself does not deplete vitamin D (221). The authors argue that as a whole, their data supports a causal role for vitamin D deficiency in COVID-19 (221).

Meltzer et al., analyzed data from their US facility's COVID-19 positive patients with documented 25(OH)D levels in EPIC within the previous 2 years to determine if deficiency increases COVID-19 incidence (222). Data for the most recent 25(OH)D and treatment (dose and time span) led to four categories (1) likely still deficient, (2) likely sufficient, (3) likely deficient but improved since testing, and (4) uncertain status (222). Known risk factors and factors that influence vitamin D activation were evaluated (222). A multivariate analysis found that, of patients with 25(OH)D levels within the previous year, those likely to still be deficient (category 1) were more likely (RR = 1.77, *p* < 0.02) to test positive for COVID-19 than those likely to be vitamin D sufficient (category 2) (222) Older age, non-white race, and immunosuppression were the only other factors associated with testing positive for COVID-19 (222). Hypertension, obesity, and diabetes were not covariates with vitamin D (222). Vitamin D deficiency was associated with supplement type and dose (*p* < 0.01), unless the relatively few patients receiving 2,000 IU or more of vitamin D_3_ were omitted (indicating that lower doses, D_2_, and calcitrol did not improve deficiency) (222). The authors concluded that the relatively low doses of vitamin D usually given to correct deficiency in their institution decreased the apparent benefit of supplementation on COVID-19 rates, and that 4,000–5,000 IU/day may be indicated for COVID-19 prevention (222).

#### Retrospective Chart Reviews That Are Neutral or Strongly Oppose the Hypothesis

Fox and Sizemore evaluated the Electronic Health Records of over 15,000,000 patients in EPIC across 26 US states, finding 28,185 patients with documented 25(OH)D (of which, 86% were deficient) and a documented COVID-19 test (85). No association was found between vitamin D deficiency (defined by each lab) and COVID-19 rates, hospitalizations, or fatalities (85). In contrast with the study by Meltzer et al., no date limits were placed on the testing; the authors noted that vitamin D levels are usually drawn to confirm suspected deficiency (85). The authors recognized this limitation and recommended future studies including patients with normal vitamin D levels, along with studies to assess the effect of vitamin D supplementation on prevention or treatment of COVID-19 (85).

Hastie et al., evaluated data from the 1,474 participants in the UK Biobank study whose COVID-19 test results were available to them (13). Rather than comparing the 1,025 PCR-negative participants to the 449 PCR-positive patients, every person in the 348,598 database without a PCR-positive test result was assumed negative (13). The 25(OH)D levels obtained 10–14 years prior were significantly lower in blacks and South Asians (13). Black or South Asian ethnicity was also strongly associated (*p* < 0.001) with confirmed COVID-19 infection (13). Median 25(OH)D was significantly lower (*p* = 0.013). for those with confirmed COVID-19 infection, and 25(OH)D predicted infection univariably (13). In contrast, the multivariate analysis did not find 25(OH)D was significant (13). Unlike most other studies of COVID-19, the authors found no association between diabetes or hypertension and COVID-19 risk, raising concerns that important variables were factored out in their analysis (13, 84).

Another review using 2006–2010 data from the UK Biobank was conducted by Darling, et al., who compared the vitamin D status, BMI, ethnicity, and lifestyle factors of 580 COVID-19 positive cases (including outpatients) with 723 negative controls of similar age (76). 25(OH)D levels were 3.6 ng/ml lower (*p* < 0.001) in patients who were obese and 6.4 ng/ml lower for those whose ethnicity was not white (*p* < 0.001) (76). COVID-19 risk was increased for non-smokers, London dwellers, males, and non-whites (76). After factoring out overweight and obesity (the factor with the highest odds ratio), and after grouping participant data into quartiles rather than using individual data, 25(OH)D did not independently predict COVID-19 risk (76).

Raisi-Estabragh et al., conducted a third multivariate analysis on the UK Biobank participants, including all 4,510 who had positive (1,326) or negative (3,184) COVID-19 tests from 16 March to 18 May 2020, almost all of whom were hospitalized (77). The researchers used the baseline data from 10 to 14 years ago for age, sex, deprivation, BMI, and 25(OH)D levels, adjusting the 25(OH)D levels for seasonality and ethnicity (77). Compared with the 497,996 untested participants of the UK Biobank study, men and non-white ethnicities were over-represented in the test group, with black ethnicity being 3.5 times more likely to be test positive than the untested cohort (77). Men and whites had higher average 25(OH)D levels than women and non-white ethnicities (77). Evaluating data from males and females independently, statistical significance was reached for males only for non-white ethnicity, more deprivation, and higher BMI (77). For women, in addition to these three factors, lower 25(OH)D, more overcrowding, and greater risk-taking were all statistically significantly related to testing COVID-19 positive (77). Rather than conducting a multivariate analysis on all potential influencers of COVID-19 positivity, Raisi-Estabragh et al., grouped exposures, testing each group against sex, age, and ethnicity, finding no significant association between these three factors, seasonally and ethnically adjusted 25(OH)D levels, and positive COVID-19 status (77). The researchers found that 25(OH)D and COVID-19 status are confounded by ethnicity and BMI (77) Mean 25(OH)D levels for both COVID-19 negative (14.18 ng/ml) and COVID-19 positive (13.55 ng/ml) primarily hospitalized patients were extremely low (77).

#### Rapid Systematic Review and Meta-Analysis With an Ecological Approach

Ghasemian et al., conducted a formal systematic review of nine studies, with six studies entering into a meta-analyses, and added their own evaluation of the correlation between global vitamin D status and COVID-19 recovery and mortality (70). The meta-analysis revealed that 46.5% of COVID-19 patients were vitamin D deficient and an additional 43.3% were vitamin D insufficient (70). Although their basic evaluation of 51 countries did not find a significant correlation between population vitamin D status and recovery or mortality rates, when latitude was factored in, both mortality rates and recovery rates weakly supported the vitamin D hypothesis (70). The researchers recommended large randomized clinical trials of vitamin D during the “Age of COVID-19” (70).

## COVID-19-Specific Recommendations of Experts

**Figure 8:**
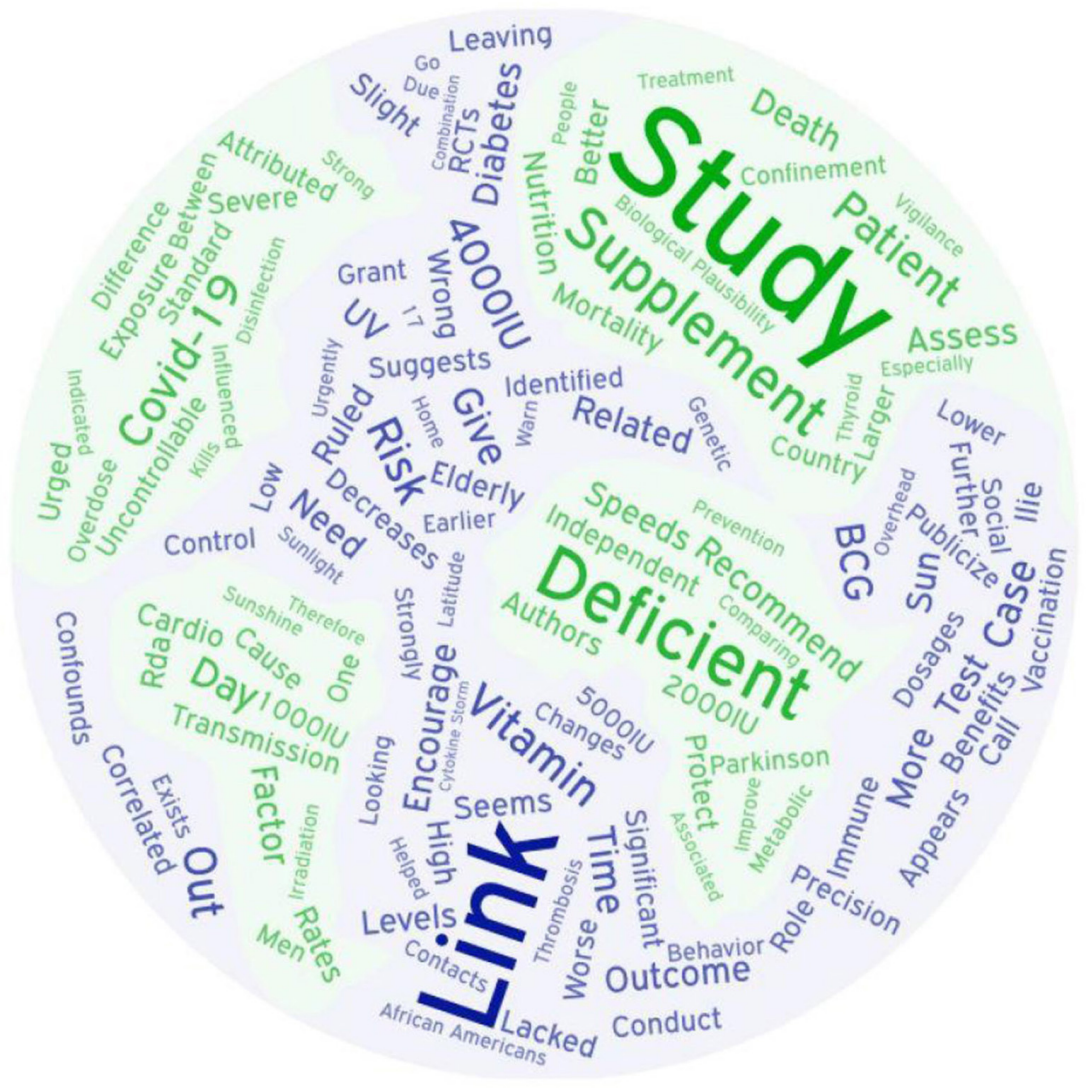
Word Cloud of recommendations from authors of 47 studies.

Although a few recommended only sunshine or 400 IU/day, none of the authors strongly opposed vitamin D supplements during the pandemic. At the extremes, some researchers recommend large bolus doses of vitamin D, or correction of deficiency, primarily for patients who are diagnosed with COVID-19, and others recommended only the dose of vitamin D needed to maintain bone health (200–400 IU/day) (44, 45, 73, 91, 108, 118, 160, 169, 223–229). Additional authors recommend vitamin D supplements to boost the immune systems of patients diagnosed with COVID-19 (2^p^, 35, 69, 71, 74, 75, 118, 120, 125, 230–233). However, most authors recommend widespread daily vitamin D supplementation (most often with 1,000–5,000 IU per day) to prevent and decrease the severity of COVID-19, at least until the pandemic abates (1, 5, 12, 28, 34, 36, 42, 64, 65, 107, 109, 113, 114, 116, 121, 124, 127, 165, 171, 172, 178, 190, 192, 197, 198, 210, 214, 219, 222, 234–248) (Figure 8).

Although vitamin D toxicity is extremely rare, considering the recent spate of chloroquine overdoses due to panic from COVID-19, recommendations include cautioning the public that excessive artificial supplementation can lead to serious harm (94, 225, 249, 250). Suresh noted that in India, vitamin D deficiency is due in large part to calcium deficiency, which must therefore also be addressed (234).

Serum response to vitamin D supplementation is highly variable between individuals, leading to recommendations of higher doses than the US RDA (28, 30). The NIH states that vitamin D supplements of up to 5,000 IU/day have not produced toxicity, leading to a maximum recommended intake for persons 9 years and older of 4,000 IU (100 mcg)/day (125, 250). Although the USRDA for vitamin D is 600–800 IU/day, the Endocrine Society and many other experts recommend 1,000–2,000 IU/day (widely available dosages) (31, 64, 65, 250). A comprehensive article on optimizing nutrition to protect against COVID-19 specifically suggests adults take 2,000 IU/day of supplemental vitamin D, in keeping with the recommendations of the US National Academy of Medicine (36, 251). The consensus of the authors reviewed here seems to be 2,000 IU/day for the entire adolescent and adult population.

## Discussion

Prior to modern times, individuals living in high latitudes had a much larger food supply from April to October, leading to weight gain (252). Excess vitamin D from sunshine was stored in accumulated fat (24, 206). Weight loss during relatively dark, food-scarce winters, released this excess vitamin D, preserving immune function (24, 206). Now, food is plentiful year-round, leading to weight gain from decreased activity in winter (252). Without weight-loss related vitamin D release, dangerously low 25(OH)D can develop by spring, and the obese, the elderly, those with naturally melanin-rich skin living outside the tropics, and anyone not spending time in the sun are at risk year-round (206).

Sunscreen with a rating of only 15 SPF decreases vitamin D production in the skin by 99% (206). Studies show that non-burning sun exposure increases vitamin D levels and may be melanoma-protective (37). In tropical areas with wealthier populations, sun exposure may decrease in the summer due to a preference for air conditioning (253). Encouraging uninfected people, including the homeless, to stay indoors could cause an increase in COVID-19 fatalities by increasing vitamin D deficiency rates. In contrast, encouraging weight loss through increased activity and structured programs can serve to improve vitamin D levels (206). Studies show that exercise increases serum vitamin D levels, even when indoors, perhaps by triggering release of vitamin D stored in fat (254).

## Conclusion

The 141 articles (Table A1) presenting primarily biological plausibility evidence overwhelmingly support the assertions that vitamin D sufficiency increases resistance to viral infections and helps prevent every symptom of severe COVID-19 that results in fatalities. They show that vitamin D deficiency can also explain every major risk factor, including the mystery of why children seem relatively protected and why males, the elderly, and people with naturally melanin-rich skin are especially vulnerable.

The 47 studies (Table A2) summarized here demonstrate that vitamin D deficiency explains the geographical differences in COVID-19 case and fatality rates. They provide overwhelming correlational evidence for the hypothesis, and causal evidence as well. COVID-19 mortality was predicted by vitamin D in 16 studies (4, 14, 24, 27, 63, 122, 127, 146, 150, 182, 197, 206, 209–211, 219) and vitamin D levels or sunlight predicted contracting COVID-19 in 17 (34, 122, 124, 150, 197, 198, 200, 201, 204, 205, 207–209, 211, 216, 221, 222). Both causal modeling studies and eight chart reviews demonstrated that lower 25(OH)D was linearly associated with more severe COVID-19 outcomes (3–5, 63, 157, 211, 215, 217, 219, 221).

None of the four objections to recommending universal vitamin D supplements are supported by the evidence. The exhaustive literature search found no vitamin D proponent who suggested that COVID-19 could be completely eliminated with supplementation. Rather than overstating the case, they present compelling evidence that vitamin D deficiency is one factor which increases risk for COVID-19 infection and progression. Although overdoses are theoretically possible, they are highly improbable. The recommended dose by consensus, 2,000 IU/day for adults, is 1/20th the amount that must be taken for many months to risk toxicity (28, 88, 91, 92). The evidence strongly suggests that vitamin D deficiency is an easily modifiable risk factor and correcting it is potentially life-saving. Suppressing this evidence out of fear that the public might believe supplements will make them “immune” to COVID-19 is not only elitist, but it is inconsistent with existing public policy approaches. Many mitigation strategies are publicized. None are seen as conferring immunity.

This succinct but comprehensive review of the evidence found that despite almost complete absence of official government guidelines favoring vitamin D supplements to potentially decrease COVID-19 risk and severity, support among clinicians and other researchers for correcting and preventing vitamin D deficiency with modest daily vitamin D supplementation during the COVID-19 pandemic is very strong, worldwide. The evidence supports recommending 2,000 IU (50 mcg) vitamin D daily for at-risk teens and adults, which is well within safe limits and might dramatically reduce COVID-19 fatalities.

## Limitations

Many of the articles and studies included in this review were preprints, or were published in haste. The study descriptions were often too brief for a critical appraisal of the designs. Definitions of variables, such as race and ethnicity, were often omitted. Many researchers did not make their data public, although some emailed corrections or clarifications. Although the author is familiar with inflammation and cytokines from her work with chronic wounds, and she is familiar with epidemiology from her health education work in developing countries, she is not an endocrinologist or an epidemiologist. Single authorship could also be considered a limitation.

## Data Availability Statement

The original contributions presented in the study are included in the article/Supplementary Material, further inquiries can be directed to the corresponding author/s.

## Author's Note

Linda Benskin is an independent nurse researcher working to improve the evidence base for village health workers in remote and conflict areas of tropical developing countries, where health care professionals are absent. Her research into how pain and inflammation impact wound healing has provided her with a basic familiarity with cytokine pathophysiology. Dr. Benskin's improvised wound dressings clinical research study has been sidelined by the travel restrictions of COVID-19. Dr. Benskin is also the Clinical Research, Education, & Charity Liaison for Ferris Mfg. Corp. (makers of PolyMem dressings).

## Author Contributions

The author confirms being the sole contributor of this work and has approved it for publication.

## Conflict of Interest

LB is employed by Ferris Mfg. Corp, makers of PolyMem dressings, and declares that the research was conducted in the absence of any commercial or financial relationships that could be construed as a potential conflict of interest.

## Footnotes

^1^Note: Because this is such a rapidly evolving topic, some of the references for this article were still in the preprint stage of publication when this review was finalized. In addition, three studies (3–5) from Southeast Asia, all with results consistent with that of other studies, may have relied upon data from unofficial sources.

## Abbreviations

RCT: Randomized Controlled Trial
25(OH)D: serum vitamin D level
RAS: Renin-Angiotensin System
UV: ultra violet (light)
UVI: ultra violet light index.
