# Supplementary material for: A Basic Review of the Preliminary Evidence That COVID-19 Risk and Severity Is Increased in Vitamin D Deficiency
Source: Front Public Health. 2020 Sep 10;8:513. doi: 10.3389/fpubh.2020.00513 (PMC7513835; doi:10.3389/fpubh.2020.00513)
Supplement: Supplementary file 1 [file Table_1.pdf]

**Table A1: In Vitro Studies and Brief Perspectives with Summaries of Pertinent Previously Published Data (141 entries)**			
1st Author /Location	Title (Truncated)	Supporting Information* /# of pages	Recommendation for Covid-19 & D (dose-who-why)
Adams/ USA	Myth Busters: Dietary Supplements and COVID-19(1)	R,J,C 7 pages	600-4000IU/day – all adults - R
Alabada/ Iraq	Vitamin D effectiveness and pathology in humans and domestic animals(2)	C 15 pages	None - Covid-19 barely mentioned
Álvarez-López /Mexico	Vitamin D y la pandemia por Covid-19 (Vitamin D and the pandemic of Covid-19)(3)	P,R,F,J,C 6 pages	10- 25 mcg/day - high risk – D, Goal = 30 ng/ml
Anderson/ UK & USA	Covid-19 Pathophysiology: Interactions of Gut Microbiome, Melatonin, Vitamin D, Stress, Kynurenine… Nicotinic … Treatment Implications(4)	P,R,F,J,C,S 24 pages	Vitamin D supplements - no details provided
Annweiler /France & China	Counter-regulatory 'Renin-Angiotensin' System-… Drugs to Treat COVID-19 …infected patients(5)	F,C,S 2 pages	RAS regulators to treat Covid-19 - no vitamin D mention
Alpalhão/ Portugal	SARS-CoV-2 pandemic and Vitamin D deficiency—A double trouble(6)	D,R,F 2 pages	20-40 min sun/day; 10-50mcg/day; test (more if deficient)
Arboleda-Alzate/ Columbia	Vitamin D supplementation: a potential approach for COVID-19 therapeutics(7)	G,R,F,C,S 9 pages	"Conventional" oral vitamin D supplementation
Ardiaria/ Indonesia	Peran vitamin d dalam pencegahan influenza dan covid-19(8)	P,R,C,J 7 pages	Clinical trials are needed urgently
Arya/ India	Synergistic effect of Vitamin D and Remdesivir can fight COVID-19(9)	R,C, 4 pages	D plus Remdesivir should be trialed
Aslan/ Turkey	Is vitamin D one of the key elements in Covid-19 days?(10)	D,G,P,R,C,S 2 pages	Supplement entire population
Aygun/ Turkey	Vitamin D can prevent COVID-19 infection-induced multiple organ damage(11)	G,R,C,S 4 pages	Supplement due to R,F
Bakare/ UK	A cost-effective preventative approach to …save lives …using Vitamin D, Curcumin, … Vitamin C(12)	R,C, 7 pages	Supplement with no added calcium
Barazzoni/ Italy, Israel, Germany, Denmark, Russia, Croatia	ESPEN expert statements and practical guidance for nutritional management of individuals with SARS-CoV-2 infection(13)	R 9 pages	Screen at-risk populations and patients for malnutrition, including vitamin D, and supplement accordingly
Barron/ UK	Vitamin D: A rapid review of the evidence for treatment or prevention of COVID-19 in adults (excluding pregnant & breastfeeding women)(14)	P,R 10 pages	Those at risk of deficiency should take 400IU/day
Bauer/	What is the role of supplementation with	D,P,R,C,	No known risks,

USA	ascorbic acid, zinc, vitamin D, or N-acetylcysteine for prevention or treatment of COVID-19?(15)	3 pages	but recommend D only after RCTs
Belančić /Croatia	Potential pathophysiological mechanisms leading to increased COVID-19 … in obesity(16)	D,P,R,C, 3 pages	Measure D and supplement obese
Biesalski/ Germany	Vitamin D deficiency and co-morbidities in COVID-19 patients – A fatal relationship?(17)	D,G,P,R,F,C,J, T,S 13 pages	D prevents storm Supplement
Bloukh/ UAE, India	Prevalence of COVID-19: A Look behind the Scenes(18)	D,P,R,C 18 pages	Prophylactically supplement if low
Brown/ UK, India	Vitamin D deficiency: a factor in COVID-19 progression severity and mortality? An urgent call for research(19,20)	D,G,P,R,F,C 29 pages	Urgent call for research, possible supplementation
Brown / UK	Rapid Response: Re: Preventing a covid-19 pandemic - COVID-19: Vitamin D deficiency; and, death rates; … connection? Research urgently…(21)	D,G,P 4 pages	Urgent call for research, possible supplementation
Brown/ UK, India, Philippines, Austria, France, Australia, Ireland, Canada, Italy, USA, Sweden, Greece, Turkey	COVID-19 ICU risk – 20-fold greater in the Vitamin D Deficient… Sun and D-supplementation – Game-changers? Research urgently required(22)	P,R,C,J,S 6 pages	Urgent call for research, correct deficiencies
Buttriss/ UK	Is a vitamin D fortification strategy needed?(23)	D,R 8 pages	400IU/day due to staying indoors
Caccialan-za/ Italy	Early nutritional supplementation in non-critically ill patients hospitalized for …(COVID-19): protocol(24)	R,C 5 pages	Correct deficiency aggressively
Calder/UK, USA, New Zealand, The Neth-erlands	Optimal Nutritional Status for a Well-Functioning Immune System Is an Important Factor to Protect against Viral Infections(25)	D,R, 10 pages	All adults should take 2000 IU of vitamin D/day, US National Academy recommendations
Cao/China , France	SARS-CoV-2 & Covid-19: Key-Roles of the 'Renin-Angiotensin' System / Vitamin D Impacting …(26)	P,R,F,C,S 2 pages	Take note: vitamin D is crucial
Carter /USA	Considerations for Obesity, Vitamin D, and Physical Activity Amid the COVID-19 Pandemic(27)	P,R,C,S 2 pages	Encourage outdoor activities, give D
Chakhtoura/Italy, USA, Lebanon	Commentary: Myths and facts on vitamin D amidst the COVID-19 pandemic(28)	G,D,R,C, 3 pages	Supplement with 1000-4000IU/day to achieve >30ng/ml 25(OH)D
Chan/ Hong Kong	What can we do for the Personal Protection against the CoVID-19 infection? Immuno-boostering(29)	R,C 6 pages	Supplement with vitamin D, herbals

Chhetri/ China, Indonesia, Japan, Korea, Thailand, Sri Lanka	Prevention of covid-19 in older adults: A brief guidance from The International Association for Gerontology and Geriatrics (IAGG) Asia/Oceania Region(30)	R<br>2 pages	Get enough sunlight "in the morning" for vitamin D production
Crane-Godreau/ USA	Vitamin D Deficiency and Air Pollution Exacerbate COVID-19 Through Suppression of Antiviral Peptide LL37(31)	D,G,P,R,F,C,<br>5 pages	Deficiency + air pollution is trouble<br>Daily dose is best
Dasgupta / India	Nsp7 and Spike Glycoprotein of SARS-CoV-2 …Potential Targets of Vitamin D and Ivermectin(32)	R,C,<br>13 pages	D may dock onto virus, be curative
De Lucena /Brazil	Mechanism of inflammatory response in associated comorbidities in COVID-19(33)	R,C,S<br>4 pages	D can decrease complications
Dhillon/ UK	COVID-19 breakthroughs: separating fact from fiction(34)	P,R,<br>37 pages	Evidence is weak<br>But, supplement
Docea/ USA, Greece, Romania, Russia	A new threat from an old enemy: Re-emergence of coronavirus (Review)(35)	R,C<br>13 pages	Evidence is weak, but biologically plausible Try D for prevention and treatment
Ebadi/ Canada	Perspective: improving vitamin D status in the management of COVID-19(36)	G,P,R,C,S<br>4 pages	Very plausible<br>High doses for pts.
Ekiz/ Turkey	Relationship between COVID-19 and obesity(37)	P,R,C<br>3 pages	Plausible link<br>Supplement
Ekiz/ Turkey	Revisiting vitamin D and … exercises for patients with sleep apnea … the COVID-19 quarantine(38)	P,R<br>6 pages	10,000IU/day x1mo then 5000IU/day
Fabbri/US A, Italy	Editorial – Vitamin D status: a key modulator of innate immunity and natural defense …(39)	D,R,C<br>5 pages	Up to 6000IU/day<br>Goal: 30 - 60ng/ml
Facchiano /Italy, USA	Reply to Jakovac: About COVID-19 and vitamin D (In support of Jakovac)(40)	R,C,S<br>1 page	No doubt<br>Conduct trials, give
Farias/ Brazil	Vitamin D as an Immunological Factor in Combating COVID-19(41)	D,P,R,C<br>10 pages	Immune benefits<br>Eat fish and eggs
Froum/ USA	Vitamin D and COVID 19 disease severity: An old ally in a new war(42)	R,C,J,<br>5 pages	Dentists should test, supplement
Garami/S witzerland	Re: Preventing a covid-19 pandemic - Is there a magic bullet to save patients?… give it a try!(43)	D,G,C,S<br>3 pages	Biological plausibility, give D!
Garg/UK, Australia	Editorial: low population mortality from COVID-19 … latitude 35 degrees North—supports vitamin D as a factor determining severity. Authors' reply(44)	D – denies others<br>2 pages	Premature, but for musculoskeletal health, give D
Gasmi/ Iran, Pakistan, Norway, Thailand,	Individual risk management strategy and potential therapeutic options for the COVID-19 pandemic(45)	D,P,R<br>9 pages	vitamin D prevents infections when given prior to exposure<br>Goal 40-60ng/ml

Luxembourg, France			10,000IU/day x1mo then 5000IU/day
Gee/UK	Management of clozapine treatment during the COVID-19 pandemic(46)	D,P,R,C<br>10 pages	D decreases risk<br>Supplement
Ghavidel-darestani/ Iran	Role of vitamin D in pathogenesis and severity of COVID-19 infection(47)	D,P,R,C,S<br>16 pages	Biological plausibility<br>D for all deficient
Glinsky/ USA	Tripartite Combination of … Pandemic Mitigation Agents: Vitamin D, Quercetin, and Estradiol(48)	D,R,C,S<br>26 pages	The 3 listed agents should be given
González/ Puerto Rico	2020 Covid-19 Protocol(49)	R,F<br>9 pages	Support immune system: 10,000IU/d<br>Infected: 25,000IU
Grant/ USA, Hungary	Evidence that Vitamin D Supplementation Could Reduce Risk of Influenza and COVID-19 Infections And Deaths(50)	R,F,C,S<br>(full review)<br>19 pages	Evidence is strong<br>Goal:40-60ng/ml<br>2000-5000IU+ daily
Grant/ USA, Hastie/UK	Letter in response to the article: Vitamin D concentrations and COVID-19 infection in UK biobank (Hastie et al.) and Response to letter(51)	Debate - see section 1.1<br>2 pages	Researchers of differing views fail to communicate
Grant/USA	Reply: "Vitamin D Supplementation in Influenza and COVID-19 Infections. Comment on: Evidence …(52)	See section 1.2, 5 pages	Response to Kow softened D dose
Guan/USA, China, Australia, Brazil, UK, Korea, South Africa, Sweden, Morocco, Russia, Mexico, Canada, Netherlands, Chile	Promoting healthy movement behaviours among children during the COVID-19 pandemic(53)	Vitamin D mentioned in passing only, assumed to be beneficial<br>3 pages	Children need to play outdoors
Gupta/ India	Is Immuno-modulation the Key to COVID-19 Pandemic?(54)	R,C<br>4 pages	D helps immunity<br>BCG vaccine
Hamad/ Sudan	Vitamin D Supplements Improve Efficacy of Minocycline…Triple Therapy to COVID-19 …(55)	R,C<br>2 pages	Improves immune<br>Add D to treatment
Hasan/Saudi Arabia, Qatar, UAE, Iran, Iraq	A review on the cleavage priming of the spike protein on coronavirus by angiotensin-converting enzyme-2 and furin (56)	S<br>9 pages	D can inhibit ACE-2 therefore could be therapeutic
Heiser/ USA	Identification of potential treatments for COVID-19 through artificial intelligence-enabled …analysis of	P,C<br>15 pages	Weak effect in D analogues – study

	human cells infected with SARS-CoV-2(57)		for new drugs
Hossain/Bangladesh	Natural Polyphenol … Crops based Diet to Promote Innate Immunity to Combat Covid-19 Disease(58)	R,C<br>22 pages	Polyphenols + D enhance immunity
Hribar/UK, USA	Potential Role of Vitamin D in the Elderly to Resist COVID-19 and to Slow … Parkinson's Disease(59)	D,P,R,F,C,J,S?<br>8 pages	Enhances immunity 2000-5000IU/day
Isaia / Italy	Possible prevention and therapeutic role of vitamin D in … the COVID-19 2020 pandemics(60)	D,G,P,R,C,J,	Enhances immunity<br>Lit. review only
Islam /Germany	A transcriptome analysis identifies potential preventive 1 and therapeutic 2 approaches …(61)	C,<br>21 Pages	Improves immune<br>Give D + ketogenic
Jakovac /Croatia	COVID-19 and vitamin D—Is there a link and an opportunity for intervention?(62)	R,C<br>1 page	D link is intriguing<br>D now – no harm
Jayawardena/Greece Sri Lanka, Australia	Enhancing Immunity in Viral Infections, with special emphasis on COVID-19: A Review(63)	D,R,C,<br>59 pages	supplementation of vitamins for Covid-19 is appropriate, especially D 5000IU
Jukic/ Croatia, Spain,	Strategies and Solutions for Team Sports Athletes in Isolation due to COVID-19(64)	No details<br>9 pages	Educate about nutrition<br>Give D
Kakodkar/ Ireland	A Comprehensive Literature Review on the Clinical Presentation, and Management … (COVID-19)(65)	R,<br>18 pages	Prophylaxis and treatment
Kalantar-Zadeh/ USA	Impact of Nutrition and Diet on COVID-19 Infection ..Implications for Kidney Health …(66)	No details<br>4 pages	Improves overall health
Kamani/ Iran	Effect of Low Level Yellow Laser Light 589nm on Virus Prevention Corona Virus (COVID-19)(67)	R<br>4 pages	Use their device to increase D
Khalil/Bangladesh	Sub-continental Atmosphere and Inherent Immune System … Impact …Covid-19… South East Asia(68)	R<br>9 pages	Influences immune<br>Get vitamin D (sun)
Khunti/UK, India	Is ethnicity linked to incidence or outcomes of covid-19?(69)	P<br>3 pages	D one of many possible reasons
Koenig/ USA, Iran, China, Saudi Arabia	Ways of Protecting Religious Older Adults from the Consequences of COVID-19(70)	P,R<br>9 pages	D is often deficient in elderly, needed for immunity<br>Take supplements
Kow/ UK, Malaysia	Vitamin D …in Influenza and COVID-19 Infections Comment on: "Evidence that Vitamin D …"(71)	Rationale OK<br>2 pages	High dose lacks evidence – trials 1st
Kumar/ India	Letter: does vitamin D have a potential role against COVID-19? (Comment on Tian, et al.)(72)	R,C,S<br>2 pages	D has two roles
Kurthkoti/ India	Potential benefits of Vitamin D supplementation in COVID-19 patients. A Hypothesis(73)	G,R,C<br>4 pages	Influences immune<br>Supplement all
La Vignera / Italy	Sex-Specific SARS-CoV-2 Mortality: Among Hormone-Modulated ACE2 Expression, Risk of … Thromboembolism and Hypovitaminosis D(74)	D,P,C,T,S<br>6 pages	D explains high male mortality & thrombosis
Lanham-New/	Vitamin D and SARS-CoV-2 virus/COVID-19 disease(75)	D,P,R,<br>5 pages	D is important, but risks are great

USA, UK			RDA or max 4000IU
Lawton/ USA	Will warmer spring weather slow down the rate of spread?(76)	G,R<br>1 page	D helps immunity
Lee /UK	Vitamin D:A rapid review of the evidence for treatment or prevention in Covid-19(77)	D,G,P,R<br>10 pages	No evidence found<br>Take RDA
Manson/ USA	Does Vitamin D Protect Against COVID-19?(78)	R,P,C,<br>1 page	Strong evidence<br>1000-2000IU
Mansur/ USA	Letter: low …mortality from COVID-19 in countries south of latitude…vitamin D …(reply to Rhodes) (79)	R,C,S<br>1 page	Additional info<br>Supports vitamin D
Martín Giménez/ Argentina	Lungs as target of COVID-19 infection: Protective … molecular mechanisms of vitamin D and melatonin … new potential synergistic treatment(80)	D,P,R,J,C,S<br>8 pages	Detailed biological plausibility data<br>D + melatonin
Maruta/ Australia	PAK1-blockers: Potential Therapeutics against COVID-19(81)	S<br>6 pages	Use MART-10, a derivative with D3
McCartney / Ireland	Optimisation of Vitamin D Status for Enhanced Immuno-protection Against Covid-19(82)	D,P,R,C<br>4 pages	800-2000IU/day - all adults - R
McCartney /Ireland	Authors of Article 'Optimisation of Vitamin D Status … comment on response … by McKenna et al(83)	D,P,<br>5 pages	800-2000IU/day - all adults - R
McCartney /Ireland	Authors of Article 'Optimisation of Vitamin D Status … comment on response … by Rabbitt et al(84)	D,R,<br>3 pages	Biological Plausibility
McCullough/ USA	The Essential Role of Vitamin D in the Biosynthesis …Antimicrobial Peptides May Explain …Deficiency Increases Mortality Risk in COVID-19 Infections(85)	G,P,R<br>23 pages	A plethora of data from other illness<br>10,000IU+/day
McKenna/ Ireland	Covid-19, Cocooning and Vitamin D Intake Requirements(86)	D,P,R<br>6 pages	NOT 800-2000IU!!!<br>Goal: 10ng/ml
Meftahi/ Iran	The possible pathophysiology mechanism of cytokine storm in elderly adults with COVID-19 infection: the contribution of "inflame-aging"(87)	P,R,F,C,S<br>15 pages	Deficiency is a likely cause of cytokine storm
Merow/ USA	Seasonality and uncertainty in COVID-19 growth rates(88)	R<br>27 pages	Increases resistance
Misra/ UK, India, Ukraine	Rheumatologists' perspective on coronavirus disease 19 (COVID-19) and potential therapeutic targets(89)	R,F,C<br>8 pages	Prevent & mitigate<br>High dose to correct if <50ng/ml
Mitchell/ UK	Vitamin-D and COVID-19: do deficient risk a poorer outcome?(90)	R,C,S<br>1 page	Influences immune<br>Correct deficiency
Molloy/ Ireland	Vitamin D, Covid-19 and Children(91)	D,G,P,R,C<br>3 pages	Influences immune<br>Correct deficiency
Muscogiuri / Italy	Nutritional recommendations for CoVID-19 quarantine(92)	D,R,J,C<br>2 pages	Change diet and eat D-rich foods
Muscogiuri / Italy	Obesity: the "Achilles heel" for COVID-19?(93) (obesity research study, vitamin D in discussion)	D,P,R,C<br>12 pages	Obesity-related D deficiency fatal?
Musselwhite / UK	JTH editorial v17 – The importance of psychosocial factors in transport and health(94)	No mention<br>2 pages	Higher D with public transport
Nigro/ Italy,	Molecular mechanisms involved in the positive effects of physical activity on coping with COVID-	P,R,J,C,<br>32 pages	Encourage physical activity, following

China, UK, Denmark	19(95)		guidelines. This will increase D
Nikhra / India	Identifying Patterns in Covid-19: Morbidity, Recovery, and the Aftermath(96)	P,R,F<br>9 pages	D supplementation may help prevent
Nizami/ India	Strong Immunity- A Major Weapon to Fight against Covid-19(97)	R,<br>8 pages	Correct deficiency for immune health
Pal / India	Managing common endocrine disorders amid COVID-19 pandemic(98)	G,P,R,C,F,S<br>5 pages	Speculation; Bone health: 800-1000IU
Palmer / Italy, UK, Sweden, Finland, Spain, Switzerland, France, Greece	The potential long-term impact of the COVID-19 outbreak on patients with non-communicable diseases in Europe: consequences for healthy ageing(99)	P,R,<br>6 pages	D deficiency is a concern; immune
Parvin/Bangladesh	The symptoms, contagious process, prevention and post treatment of Covid-19(100)	P<br>25 pages	Sunlight and daily supplements
Pinnock/ UK	Vitamin D and COVID-19. The Evidence Warrants Discussion!(101)	G,R,C<br>5 pages	Immune benefits 1000-2000IU
Queiroz / Brazil	Management of inflammatory bowel disease patients …COVID-19 pandemic…: a Brazilian … (102)	R<br>6 pages	Immune benefits Always supplement
Quesada-Gomez/ Spain, Belgium	Vitamin D Receptor stimulation to reduce Acute Respiratory Distress Syndrome (ARDS) in patients with Coronavirus SARS-CoV-2 infections(103)	D,P,R,C,T,S,<br>23 pages	Prevents infection Controls immune Calcifediol is best absorbed form
Rabbitt/ Ireland	Vitamin D and Covid-19: A Note of Caution (response to McCartney, Optimisation of … D)(104)	(none)<br>2 pages	D is safe but need RCTs first
Ramos/ Brazil	COVID-19, Rate of Case Factors and Nutritional Characteristics of …Dying in Italy and Brazil: …(105)	D,P,R,C,F,<br>9 pages	Immune benefits 50,000IU/week
Raymond/ UK	Mast cell stabilisers, leukotriene antagonists and antihistamines: … evidence … use in COVID-19(106)	R,C<br>6 pages	No evidence of a D Covid-19 link
Razdan / India	Vitamin D Levels and COVID-19 Susceptibility: Is there any Correlation?(107)	D,R,C<br>3 pages	Clear Covid-19 link 2000-4000IU/day
Rhodes/ UK	Letter: low population mortality from COVID-19 …south of latitude 35° North supports vitamin D as a factor ...severity—authors' reply (to Mansur)(108)	S,T<br>2 pages	Circumstantial evidence is strong South: supplement
Ribeiro/ Brazil, UK, Australia	Does Vitamin D play a role in the management of Covid-19 in Brazil? (Building upon Isaia literature review)(109)	D,P,R,<br>6 pages	Enhances Immunity Get more sun. Do not take 10,000IU
Richards/ South Africa	COVID-19 and the Rationale for Pharmacotherapy: A South African Perspective(110)	S<br>7 pages	50,000IU stat for acute treatment 4000IU/day if mild
Richer/ USA	How to build a lifestyle and nutritional firewall against viruses like COVID 19(111)	D,P,R<br>5 pages	Enhances Immunity D in multivitamin

Rocha/UK, Portugal	Reply to Jakovac; Severity of COVID-19 infection in patients with phenylketonuria: is vitamin D status protective? (In support of Jakovac)(112)	P,R<br>2 pages	Enhances Immunity<br>PKU supplement<br>Supplement all
Romano / Italy	Short Report – Medical nutrition therapy for critically ill patients with COVID-19(113)	(none)<br>5 pages	Correct <12.5ng/ml (IV cholecalciferol)
Roy/ UK	Response to 'Vitamin D concentrations and COVID-19 infection in UK Biobank' (Hastie)(114)	R,F,J,<br>1 page	Enhances Immunity<br>Better research!
Rusciano/ Italy	The Fight against COVID-19: The Role of Drugs and Food Supplements(115)	D,P,R,J,C<br>16 pages	Enhances Immunity<br>eat well
Saul/ USA	Nutritional Treatment of Coronavirus(116)	(none)<br>9 pages	5000IU/day x 2 weeks then 2000IU
Sharma/ India	Vitamin D: A cheap yet effective bullet against coronavirus disease-19 – Are we convinced yet?(117)	D,G,P,R,F,C,J, S 8 pages	Evidence is strong<br>1000-2000IU/day
Siddiqui/ UAE	Centralized air-conditioning and transmission of novel coronavirus(118)	D,R<br>3 pages	Air conditioning limits outdoor time
Silberstein / Australia	Vitamin D: A simpler alternative to tocilizumab for trial in COVID-19?(119)	D,G,P,R,C<br>2 pages	Biological plausibility – study!
Silberstein / Australia	Premorbid IL-6 levels may predict mortality from COVID-19 (studied IL-6 levels, not vitamin D) (120)	P,C<br>9 pages	Correlation strong<br>Supplement all
Siuka/ Slovenia	Vitamin D supplementation in the COVID-19 pandemic(121)	D,P,R,F,C<br>3 pages	Supplement high-risk, urgently
Sparavigna/ Italy	Vitamin D for Covid-19?(122)	D,R,C,<br>8 pages	Lists literature - no conclusion drawn
Speeckaert/ Belgium	Association between low vitamin D and COVID-19: don't forget the vitamin D binding protein(123)	C,T<br>2 pages	Decreases ARDs<br>Avoid deficiency
Suresh/ India	Hypovitaminosis D and COVID-19: Matter of Concern in India?(124)	D,G,P,R,C,S<br>2 pages	Study, and correct D deficiency
Taheri/ Qatar	Managing diabetes in Qatar during the COVID-19 pandemic(125)	R<br>2 pages	D is delivered to diabetic patients
Tan/ Singapore	Medications in COVID-19 patients: summarizing the current literature … orthopaedic perspective(126)	R,C,<br>5 pages	Continue, but don't add, B, C, and D
Thomson/ UK	The COVID-19 Pandemic: A Global Natural Experiment(127)	(none)<br>6 pages	Seasons allow study of D impact
Tian/ China	Letter: Covid-19, and vitamin D. Authors' reply (to Panarese)(128)	G,R,J,C,T,S<br>2 pages	Theory is sound<br>Supplement all
Tian/ China	Letter: does vitamin D have a potential role against COVID-19? Authors' reply (to Kumar)(129)	R,C,T,<br>4 pages	Theory is sound<br>Moderate doses
Trinity College Dublin/ Ireland	News Release 12-May-2020 Vitamin D determines severity in COVID-19 so government advice needs to change(130)	D,G,P,F,C<br>3 pages	Irish government should support supplements and food fortification
Turashvili/ Georgia	Could vitamin D reduce the risk of COVID-19?(131)	G,P,R,C,S<br>5 pages	Biological plausibility – study!
Uçar/	Koronavirüs ve fitoterapi(132)	R,C,	Boosts immunity

Turkey		9 pages	Give supplements
Wang/ China	Review of the 2019 novel coronavirus (SARS-CoV-2) based on current evidence(133)	R<br>7 pages	Boosts immunity<br>Give supplements
Weir/ USA	Does vitamin D deficiency increase the severity of COVID-19?(134)	D,P,R,C,T<br>2 pages	Boosts immunity<br>Give modest doses
Wimalawansa/ USA	Global epidemic of coronavirus—Covid-19: What can we do to minimize risks(135)	D,R,C,<br>7 pages	Loading dose for all 200,000-300,000IU
Yong/ Malaysia	Population Studies Confirm Risk Factors for Catching Covid-19(136) and Vitamin D as an Independent Risk Factor for COVID-19 Death(137)	F<br>5 & 10 pages	Studies are compelling
Yousfi/ Tunisia, Italy, Poland, Canada, Qatar	The COVID-19 pandemic: how to maintain a healthy immune … special focus on athletes.(138)	P,R,C,F<br>7 pages	10,000IU/day x1mo then 5000IU/day
Zabetakis/ Ireland, USA	COVID-19: The Inflammation Link and the Role of Nutrition in Potential Mitigation(139)	P,R<br>28 pages	Uncertain if high doses required, but supplement
Zemb/ France, Sweden, Belgium, Italy, USA, Austria, Poland, Romania	Vitamin D deficiency and COVID-19 pandemic(140)	D,P,R,<br>5 pages	Immune benefits for other infections are known, but not for Covid-19.<br>No harm found with 2000-4000IU, so why not supplement?
Zhang/ Japan, China	Current status of potential therapeutic candidates for the COVID-19 crisis(141)	R,C	Studies including D are underway (not yet recommended)
Zhang/ China	Potential interventions for novel coronavirus in China: A systematic review(142)	D,G,P,R	Could work to prevent
Zicarelli/ Canada	Nutrition Therapy … (COVID-19): Recommendations … for Integrative Medical Treatments(143)	R,C,<br>9 pages	Benefits patients 2000IU/day

* Supporting Information provided by these authors:
D = vitamin D deficiency is common
G = geographical observations (not data) of relationship between low vitamin D and high Covid-19
P = deficiency is more common in populations at higher risk for severe Covid-19 complications
Biological plausibility:
R = vitamin D enhances resistance to respiratory viruses, decreasing incidence of infection
F = vitamin D decreases overall fatalities from respiratory viruses; decreases Covid-19 severity
C = vitamin D suppresses pro-inflammatory cytokines implicated in severe Covid-19
J = vitamin D tightens junctions, helping prevent viral infections from progressing to pneumonia
T = vitamin D decreases the risk of thrombosis

S = vitamin D suppresses the ‘Renin-Angiotensin’ System activity, which is more of a problem for males

# of pages includes references, figures, and tables

References

1. Adams KK, Baker WL, Sobieraj DM. Myth Busters: Dietary Supplements and COVID-19. *Ann Pharmacother* (2020)1060028020928052. doi:10.1177/1060028020928052

2. Alabada HKM, Saleh WMM. Vitamin D effectiveness and pathology in humans and domestic animals. *Multi* (2020) **3**:15.

3. Álvarez-López JA, García-Contreras AI. Vitamina D y la pandemia por COVID-19. *RME* (2020) **7**:4329. doi:10.24875/RME.20000036

4. Anderson G, Reiter RJ. COVID-19 pathophysiology: interactions of gut microbiome, melatonin, vitamin D, stress, kynurenine and the alpha 7 nicotinic receptor: Treatment implications. *Melatonin Research* (2020) **3**:322–345. doi:10.32794/mr11250066

5. Annweiler C, Cao Z, Wu Y, Faucon E, Mouhat S, Kovacic H, Sabatier J-M. Counter-regulatory “Renin-Angiotensin” System-based Candidate Drugs to Treat COVID-19 Diseases in SARS-CoV-2-infected patients. *Infect Disord Drug Targets* (2020) doi:10.2174/1871526520666200518073329

6. Alpalhão M, Filipe P. SARS-CoV-2 pandemic and Vitamin D deficiency—A double trouble. *Photodermatology, Photoimmunology & Photomedicine* **n/a**: doi:10.1111/phpp.12579

7. Arboleda J, Urcuqui-Inchima S. Vitamin D supplementation: a potential approach for COVID-19 therapeutics? Open Science Framework (2020). doi:10.31219/osf.io/cgd4t

8. Ardiaria M. PERAN VITAMIN D DALAM PENCEGAHAN INFLUENZA DAN COVID-19. *JNH (Journal of Nutrition and Health)* (2020) **8**:79–85. doi:10.14710/jnh.8.2.2020.79-85

9. Arya A, Dwivedi VD. Synergistic effect of Vitamin D and Remdesivir can fight COVID-19. *Journal of Biomolecular Structure and Dynamics* (2020) **0**:1–2. doi:10.1080/07391102.2020.1773929

10. Aslan MT, Aslan İÖ, Özdemir Ö. Is Vitamin D One of the Key Elements in COVID-19 Days? *J Nutr Health Aging* (2020) doi:10.1007/s12603-020-1413-5

11. Aygun H. Vitamin D can prevent COVID-19 infection-induced multiple organ damage. *Naunyn Schmiedebergs Arch Pharmacol* (2020) doi:10.1007/s00210-020-01911-4

12. Bakare TA, Soar JS. A cost-effective preventative approach to potentially save lives in the coronavirus pandemic, jointly using Vitamin D, Curcumin, and Vitamin C. NutriXiv (2020). doi:10.31232/osf.io/jgf6e

13. Barazzoni R, Bischoff SC, Breda J, Wickramasinghe K, Krznaric Z, Nitzan D, Pirlich M, Singer P. ESPEN expert statements and practical guidance for nutritional management of individuals with SARS-CoV-2 infection. *Clin Nutr* (2020) doi:10.1016/j.clnu.2020.03.022

14. Barron A, Patel N. Vitamin D: A rapid review of the evidence for treatment or prevention of COVID-19 in adults (excluding pregnant & breastfeeding women). London, UK: NIH (2020). Available at: https://www.ncl-mon.nhs.uk/faq/guidelines/

15. Bauer SR, Kapoor A, Rath M, Thomas SA. What is the role of supplementation with ascorbic acid, zinc, vitamin D, or N-acetylcysteine for prevention or treatment of COVID-19? *CCJM* (2020) doi:10.3949/ccjm.87a.ccc046

16. Belančić A, Kresović A, Rački V. Potential pathophysiological mechanisms leading to increased COVID-19 susceptibility and severity in obesity. *Obes Med* (2020) **19**:100259. doi:10.1016/j.obmed.2020.100259

17. Biesalski HK. Vitamin D deficiency and co-morbidities in COVID-19 patients – A fatal relationship? *NFS Journal* (2020) **20**:10–21. doi:10.1016/j.nfs.2020.06.001

18. Bloukh SH, Shaikh AA, Pathan HM, Edis Z. Prevalence of COVID-19: A Look behind the Scenes. (2020) doi:10.20944/preprints202004.0179.v2

19. Brown RA, Sarkar A. Vitamin D deficiency: a factor in COVID-19, progression, severity and mortality? – An urgent call for research. *Modern Ghana* (2020) Available at: https://www.modernghana.com/news/992403/vitamin-d-deficiency-a-factor-in-covid-19-progre.html [Accessed April 23, 2020]

20. Brown RA, Sarkar A. Vitamin D deficiency: a factor in COVID-19, progression, severity and mortality? – An urgent call for research. *MitoFit Preprint Arch* (2020) Available at: https://www.mitofit.org/index.php/Brown_2020_MitoFit_Preprint_Arch [Accessed April 23, 2020]

21. Brown RA. Re: Preventing a covid-19 pandemic - COVID-19: Vitamin D deficiency; and, death rates; are both disproportionately higher in elderly Italians, Spanish, Swedish Somali, and African Americans? A connection? Research urgently required! *The BMJ* (2020) Available at: https://www.bmj.com/content/368/bmj.m810/rr-46 [Accessed April 23, 2020]

22. Brown RA, Rhein H, Alipio MM, Annweiler C, Gnaiger E, Holick MF, Boucher BJ, Duque G, Féron F, Kenny RA, Montero-Odasso M, Minisola S, Rhodes J, Haq A, Bejerot S, Reiss LAJ, Zgaga L, Crawford MA, Fricker RA, Cobbold P, Lahore HW, Humble MB, Sakar A, Karras S, Iglesias-Gonzalez J, Gezen-Ak D, Dursun E, Cooper I, Grimes D, de Voil CWB,, McCarrison Society, La Route de Mont Cochon, St Lawrence, Jersey. COVID-19 ’ICU’ risk – 20-fold greater in the Vitamin D Deficient. BAME, African Americans, the Older, Institutionalised and Obese, are at greatest risk. Sun and ‘D’-supplementation – Game-changers? Research urgently required. *BMJ* (2020) **2020;369:m1548/rr–6**: Available at: https://www.bmj.com/content/369/bmj.m1548/rr-6 [Accessed June 24, 2020]

23. Buttriss JL, Lanham-New SA. Is a vitamin D fortification strategy needed? *Nutr Bull* (2020) **45**:115–122. doi:10.1111/nbu.12430

24. Caccialanza R, Laviano A, Lobascio F, Montagna E, Bruno R, Ludovisi S, Corsico AG, Di Sabatino A, Belliato M, Calvi M, et al. Early nutritional supplementation in non-critically ill patients hospitalized

for the 2019 novel coronavirus disease (COVID-19): Rationale and feasibility of a shared pragmatic protocol. *Nutrition* (2020)110835. doi:10.1016/j.nut.2020.110835

25. Calder PC, Carr AC, Gombart AF, Eggersdorfer M. Optimal Nutritional Status for a Well-Functioning Immune System Is an Important Factor to Protect against Viral Infections. *Nutrients* (2020) **12**: doi:10.3390/nu12041181

26. Cao Z, Wu Y, Faucon E, Sabatier J-M. SARS-CoV-2 & Covid-19: Key-Roles of the “Renin-Angiotensin” System / Vitamin D Impacting Drug and Vaccine Developments. *Infect Disord Drug Targets* (2020) doi:10.2174/1871526520999200505174704

27. Carter SJ, Baranauskas MN, Fly AD. Considerations for obesity, vitamin D, and physical activity amidst the COVID-19 pandemic. *Obesity (Silver Spring)* (2020) doi:10.1002/oby.22838

28. Chakhtoura M, Napoli N, El Hajj Fuleihan G. Commentary: Myths and facts on vitamin D amidst the COVID-19 pandemic. *Metabolism* (2020) **109**:154276. doi:10.1016/j.metabol.2020.154276

29. Chan B, Wong C, Leung PC. What can we do for the Personal Protection against the CoVID-19 Infection? Immuno-Boostering Specific Supplement could be the Answer. *J Emerg Med Trauma Surg Care* (2020) **2**:

30. Chhetri JK, Chan P, Arai H, Chul Park S, Sriyani Gunaratne P, Setiati S, Assantachai P. Prevention of COVID-19 in Older Adults: A Brief Guidance from the International Association for Gerontology and Geriatrics (IAGG) Asia/Oceania region. *J Nutr Health Aging* (2020) **24**:471–472. doi:10.1007/s12603-020-1359-7

31. Crane-Godreau MA, Clem KJ, Payne P, Fiering S. Vitamin D Deficiency and Air Pollution Exacerbate COVID-19 Through Suppression of Antiviral Peptide LL37. *Front Public Health* (2020) **8**: doi:10.3389/fpubh.2020.00232

32. Dasgupta J, Sen U, Bakshi A, Dasgupta A, Manna K, Saha C, De RK, Mukhopadhyay S, Bhattacharyya NP. Nsp7 and Spike Glycoprotein of SARS-CoV-2 Are Envisaged as Potential Targets of Vitamin D and Ivermectin. (2020) doi:10.20944/preprints202005.0084.v1

33. de Lucena TMC, da Silva Santos AF, de Lima BR, de Albuquerque Borborema ME, de Azevêdo Silva J. Mechanism of inflammatory response in associated comorbidities in COVID-19. *Diabetes Metab Syndr* (2020) **14**:597–600. doi:10.1016/j.dsx.2020.05.025

34. Dhillon P, Breuer M, Hirst N. COVID-19 breakthroughs: separating fact from fiction. *The FEBS Journal* (2020)

35. Docea AO, Tsatsakis A, Albulescu D, Cristea O, Zlatian O, Vinceti M, Moschos SA, Tsoukalas D, Goumenou M, Drakoulis N, et al. A new threat from an old enemy: Re-emergence of coronavirus (Review). *Int J Mol Med* (2020) **45**:1631–1643. doi:10.3892/ijmm.2020.4555

36. Ebadi M, Montano-Loza AJ. Perspective: improving vitamin D status in the management of COVID-19. *Eur J Clin Nutr* (2020) doi:10.1038/s41430-020-0661-0

37. Ekiz T, Pazarlı AC. Relationship between COVID-19 and obesity. *Diabetes & Metabolic Syndrome: Clinical Research & Reviews* (2020) **14**:761–763. doi:10.1016/j.dsx.2020.05.047

38. Ekiz T, Kara M, Özçakar L. Revisiting vitamin D and home-based exercises for patients with sleep apnea facing the COVID-19 quarantine. *J Clin Sleep Med* (2020) doi:10.5664/jcsm.8586

39. Fabbri A, Infante M, Ricordi C. Editorial - Vitamin D status: a key modulator of innate immunity and natural defense from acute viral respiratory infections. *Eur Rev Med Pharmacol Sci* (2020) **24**:4048–4052. doi:10.26355/eurrev_202004_20876

40. Facchiano A, Facchiano A, Bartoli M, Ricci A, Facchiano F. Reply to Jakovac: About COVID-19 and vitamin D. *Am J Physiol Endocrinol Metab* (2020) **318**:E838. doi:10.1152/ajpendo.00185.2020

41. Farias E dos S, Silva DF, Conceição VS da, Rocha AM. Vitamin D as an Immunological Factor in Combating COVID-19. *International Journal of Advanced Engineering Research and Science* (2020) **7**: Available at: http://journal-repository.com/index.php/ijaers/article/view/2067 [Accessed June 16, 2020]

42. Froum S. Vitamin D and COVID-19 disease severity: An old ally in a new war. *Perio-Implant Advisory* (2020) Available at: https://www.perioimplantadvisory.com/clinical-tips/article/14176684/vitamin-d-and-covid19-disease-severity-an-old-ally-in-a-new-war [Accessed June 16, 2020]

43. Garami AR. Re: Preventing a covid-19 pandemic - Is there a magic bullet to save COVID-19 patients? We can give it a try! *BMJ (British Medical Journal)* (2020) **2020;368:m810**: Available at: https://www.bmj.com/content/368/bmj.m810/rr-24 [Accessed May 10, 2020]

44. Garg M, Al-Ani A, Mitchell H, Hendy P, Christensen B. Editorial: low population mortality from COVID-19 in countries south of latitude 35 degrees North - supports vitamin D as a factor determining severity. Authors' reply. *Aliment Pharmacol Ther* (2020) doi:10.1111/apt.15796

45. Gasmi A, Noor S, Tippairote T, Dadar M, Menzel A, Bjørklund G. Individual risk management strategy and potential therapeutic options for the COVID-19 pandemic. *Clinical Immunology* (2020) **215**:108409. doi:10.1016/j.clim.2020.108409

46. Gee S, Gaughran F, MacCabe J, Shergill S, Whiskey E, Taylor D. Management of clozapine treatment during the COVID-19 pandemic: *Therapeutic Advances in Psychopharmacology* (2020) doi:10.1177/2045125320928167

47. Ghavideldarestani M, Honardoost M, Khamseh ME. Role of Vitamin D in Pathogenesis and Severity of COVID-19 Infection. (2020) doi:10.20944/preprints202004.0355.v1

48. Glinsky GV. Tripartite Combination of Candidate Pandemic Mitigation Agents: Vitamin D, Quercetin, and Estradiol Manifest Properties of Medicinal Agents for Targeted Mitigation of the COVID-19 Pandemic Defined by Genomics-Guided Tracing of SARS-CoV-2 Targets in Human Cells. *Biomedicines* (2020) **8**: doi:10.3390/biomedicines8050129

49. Vázquez--Tanus JB. COVID-19 Protocol. Puerto Rico: Vascular Nutrition PR Website (4/12/20202). Available at: https://www.vascularnutritionpr.com/covid-19.html [Accessed June 22, 2020]

50. Grant WB, Lahore H, McDonnell SL, Baggerly CA, French CB, Aliano JL, Bhattoa HP. Evidence that Vitamin D Supplementation Could Reduce Risk of Influenza and COVID-19 Infections and Deaths. *Nutrients* (2020) **12**:988. doi:10.3390/nu12040988

51. Grant WB, McDonnell SL. Letter in response to the article: Vitamin D concentrations and COVID-19 infection in UK biobank (Hastie et al.). *Diabetes & Metabolic Syndrome: Clinical Research & Reviews* (2020) **14**:893–894. doi:10.1016/j.dsx.2020.05.046

52. Grant WB, Baggerly CA, Lahore H. Reply: "Vitamin D Supplementation in Influenza and COVID-19 Infections. Comment on: Evidence That Vitamin D Supplementation Could Reduce Risk of Influenza and COVID-19 Infections and Deaths Nutrients 2020, 12(4), 988." *Nutrients* (2020) **12**:1620. doi:10.3390/nu12061620

53. Guan H, Okely AD, Aguilar-Farias N, del Pozo Cruz B, Draper CE, El Hamdouchi A, Florindo AA, Jáuregui A, Katzmarzyk PT, Kontsevaya A, et al. Promoting healthy movement behaviours among children during the COVID-19 pandemic. *Lancet Child Adolesc Health* (2020) **4**:416–418. doi:10.1016/S2352-4642(20)30131-0

54. Gupta A. Is Immuno-modulation the Key to COVID-19 Pandemic? *Indian J Orthop* (2020)1–4. doi:10.1007/s43465-020-00121-7

55. Mohammed Hamad MN. Vitamin D Supplements Improve Efficacy of Minocycline, N-Acetylcysteine and Aspirin Triple Therapy to COVID-19 Infection. *SJBR* (2020) **05**:59–60. doi:10.36348/sjbr.2020.v05i04.005

56. Hasan A, Paray BA, Hussain A, Qadir FA, Attar F, Aziz FM, Sharifi M, Derakhshankhah H, Rasti B, Mehrabi M, et al. A review on the cleavage priming of the spike protein on coronavirus by angiotensin-converting enzyme-2 and furin. *J Biomol Struct Dyn* (2020) **0**:1–9. doi:10.1080/07391102.2020.1754293

57. Heiser K, McLean PF, Davis CT, Fogelson B, Gordon HB, Jacobson P, Hurst B, Miller B, Alfa RW, Earnshaw BA, et al. Identification of potential treatments for COVID-19 through artificial intelligence-enabled phenomic analysis of human cells infected with SARS-CoV-2. *bioRxiv* (2020)2020.04.21.054387. doi:10.1101/2020.04.21.054387

58. Hossain Z, Uddin MdN. Natural Polyphenol Engineering Field Crops based Diet to Promote Innate Immunity to Combat Covid-19 Disease. AgriXiv (2020). doi:10.31220/osf.io/da85c

59. Hribar CA, Cobbold PH, Church FC. Potential Role of Vitamin D in the Elderly to Resist COVID-19 and to Slow Progression of Parkinson's Disease. *Brain Sci* (2020) **10**: doi:10.3390/brainsci10050284

60. Isaia G, Medico E. Possible prevention and therapeutic role of vitamin D in the management of the COVID-19 2020 pandemics. (2020) Available at:

https://www.unitonews.it/storage/2515/8522/3585/Ipovitaminosi_D_e_
Coronavirus_25_marzo_2020.pdf

61. Islam MR, Fischer A. A Transcriptome Analysis Identifies Potential Preventive and Therapeutic Approaches Towards COVID-19. (2020) doi:10.20944/preprints202004.0399.v1

62. Jakovac H. COVID-19 and vitamin D—Is there a link and an opportunity for intervention? *American Journal of Physiology-Endocrinology and Metabolism* (2020) **318**:E589–E589. doi:10.1152/ajpendo.00138.2020

63. Jayawardena R, Sooriyaarachchi P, Chourdakis M, Jeewandara C, Ranasinghe P. Enhancing immunity in viral infections, with special emphasis on COVID-19: A review. *Diabetes Metab Syndr* (2020) doi:10.1016/j.dsx.2020.04.015

64. Jukic I, Calleja-González J, Cos F, Cuzzolin F, Olmo J, Terrados N, Njaradi N, Sassi R, Requena B, Milanovic L, et al. Strategies and Solutions for Team Sports Athletes in Isolation due to COVID-19. *Sports (Basel)* (2020) **8**: doi:10.3390/sports8040056

65. Kakodkar P, Kaka N, Baig MN. A Comprehensive Literature Review on the Clinical Presentation, and Management of the Pandemic Coronavirus Disease 2019 (COVID-19). *Cureus* (2020) **12**:e7560. doi:10.7759/cureus.7560

66. Kalantar-Zadeh K, Moore LW. Impact of Nutrition and Diet on COVID-19 Infection and Implications for Kidney Health and Kidney Disease Management. *J Ren Nutr* (2020) **30**:179–181. doi:10.1053/j.jrn.2020.03.006

67. Kamani E. Effect of Low Level Yellow Laser Light 589nm on Virus Prevention Corona Virus (COVID-19). *OSP Journal of Case Reports* (2020) **2**:1–4. Available at: https://www.ospublishers.com/Effect-of-Low-Level-Yellow-Laser-Light-589nm-on-Virus-Prevention-Corona-Virus-(COVID-19).html [Accessed June 16, 2020]

68. Khalil I, Barma P. Sub-continental Atmosphere and Inherent Immune System may have Impact on Novel Corona Virus' 2019 (nCovid-19) Prevalence in South East Asia. *Mymensingh Med J* (2020) **29**:473–480.

69. Khunti K, Singh AK, Pareek M, Hanif W. Is ethnicity linked to incidence or outcomes of covid-19? *BMJ* (2020) **369**: doi:10.1136/bmj.m1548

70. Koenig HG. Ways of Protecting Religious Older Adults from the Consequences of COVID-19. *Am J Geriatr Psychiatry* (2020) doi:10.1016/j.jagp.2020.04.004

71. Kow CS, Hadi MA, Hasan SS. Vitamin D Supplementation in Influenza and COVID-19 Infections Comment on: "Evidence that Vitamin D Supplementation Could Reduce Risk of Influenza and COVID-19 Infections and Deaths" Nutrients 2020, 12(4), 988. *Nutrients* (2020) **12**: doi:10.3390/nu12061626

72. Kumar D, Gupta P, Banerjee D. Letter: does vitamin D have a potential role against COVID-19? *Aliment Pharmacol Ther* (2020) doi:10.1111/apt.15801

73. Kurthkoti K, Das G. Potential benefits of Vitamin D supplementation in COVID-19 patients: a hypothesis. Open Science Framework (2020). doi:10.31219/osf.io/dnjav

74. La Vignera S, Cannarella R, Condorelli RA, Torre F, Aversa A, Calogero AE. Sex-Specific SARS-CoV-2 Mortality: Among Hormone-Modulated ACE2 Expression, Risk of Venous Thromboembolism and Hypovitaminosis D. *Int J Mol Sci* (2020) **21**: doi:10.3390/ijms21082948

75. Lanham-New SA, Webb AR, Cashman KD, Buttriss JL, Fallowfield JL, Masud T, Hewison M, Mathers JC, Kiely M, Welch AA, et al. Vitamin D and SARS-CoV-2 virus/COVID-19 disease. *BMJ Nutrition, Prevention & Health* (2020)bmjnph-2020-000089. doi:10.1136/bmjnph-2020-000089

76. Lawton G. Will warmer spring weather slow down the rate of spread? *New Sci* (2020) **245**:8. doi:10.1016/S0262-4079(20)30659-X

77. Lee J, van Hecke O, Roberts N. Vitamin D: A rapid review of the evidence for treatment or prevention in COVID-19. *CEBM* Available at: https://www.cebm.net/covid-19/vitamin-d-a-rapid-review-of-the-evidence-for-treatment-or-prevention-in-covid-19/ [Accessed May 4, 2020]

78. Manson JE. Does Vitamin D Protect Against COVID-19? *Medscape* (2020) Available at: http://www.medscape.com/viewarticle/930152 [Accessed May 12, 2020]

79. Mansur J. Letter: low population mortality from COVID-19 in countries south of latitude 35 degrees North supports vitamin D as a factor determining severity. *Aliment Pharmacol Ther* (2020) doi:10.1111/apt.15820

80. Martín Giménez VM, Inserra F, Tajer CD, Mariani J, Ferder L, Reiter RJ, Manucha W. Lungs as target of COVID-19 infection: Protective common molecular mechanisms of vitamin D and melatonin as a new potential synergistic treatment. *Life Sci* (2020) **254**:117808. doi:10.1016/j.lfs.2020.117808

81. Maruta H, He H. PAK1-blockers: Potential Therapeutics against COVID-19. *Med Drug Discov* (2020) **6**:100039. doi:10.1016/j.medidd.2020.100039

82. McCartney DM, Byrne DG. Optimisation of Vitamin D Status for Enhanced Immuno-protection Against Covid-19. *Ir Med J* (2020) **113**:58.

83. McCartney DM, Byrne DG. McCartney et al comment on ‘Covid-19, Cocooning and Vitamin D Requirements’ response report – Irish Medical Journal. *Irish Medical Journal* (2020) **113**:80. Available at: http://imj.ie/mccartney-et-al-comment-on-covid-19-cocooning-and-vitamin-d-requirements-response-report/ [Accessed May 14, 2020]

84. McCartney DM, Byrne DG. McCartney et al comment on ‘Vitamin D and Covid-19 – A Note of Caution’ response letter – Irish Medical Journal. *Irish Medical Journal* **113**:79. Available at: http://imj.ie/mccartney-et-al-comment-on-vitamin-d-and-covid-19-a-note-of-caution-response-letter/ [Accessed May 14, 2020]

85. McCullough PJ, Amend J, McCullough WP, Repas SJ, Travers JB, Lehrer DS. The Essential Role of Vitamin D in the Biosynthesis of Endogenous Antimicrobial Peptides May Explain Why Deficiency Increases Mortality Risk in COVID-19 Infections. (2020) doi:10.20944/preprints202005.0265.v1

86. McKenna MJ, Flynn MAT. Covid-19 Cocooning and Vitamin D Intake Requirements – Irish Medical Journal. *Irish Medical Journal* (2020) **113**:79. Available at: http://imj.ie/covid-19-cocooning-and-vitamin-d-intake-requirements/ [Accessed May 14, 2020]

87. Meftahi GH, Jangravi Z, Sahraei H, Bahari Z. The possible pathophysiology mechanism of cytokine storm in elderly adults with COVID-19 infection: the contribution of "inflame-aging." *Inflamm Res* (2020) doi:10.1007/s00011-020-01372-8

88. Merow C, Urban MC. Seasonality and uncertainty in COVID-19 growth rates. *medRxiv* (2020)2020.04.19.20071951. doi:10.1101/2020.04.19.20071951

89. Misra DP, Agarwal V, Gasparyan AY, Zimba O. Rheumatologists' perspective on coronavirus disease 19 (COVID-19) and potential therapeutic targets. *Clin Rheumatol* (2020) doi:10.1007/s10067-020-05073-9

90. Mitchell F. Vitamin-D and COVID-19: do deficient risk a poorer outcome? *The Lancet Diabetes & Endocrinology* (2020) **0**: doi:10.1016/S2213-8587(20)30183-2

91. Molloy EJ, Murphy N. Vitamin D, Covid-19 and Children. *Ir Med J* (2020) **113**:64.

92. Muscogiuri G, Barrea L, Savastano S, Colao A. Nutritional recommendations for CoVID-19 quarantine. *European Journal of Clinical Nutrition* (2020) **74**:850–851. doi:10.1038/s41430-020-0635-2

93. Muscogiuri G, Pugliese G, Barrea L, Savastano S, Colao A. Obesity: the "Achilles heel" for COVID-19? *Metabolism* (2020) doi:10.1016/j.metabol.2020.154251

94. Musselwhite CBA. JTH editorial v17 – The importance of psychosocial factors in transport and health. *J Transp Health* (2020) doi:10.1016/j.jth.2020.100876

95. Nigro E, Polito R, Alfieri A, Mancini A, Imperlini E, Elce A, Krustrup P, Orrù S, Buono P, Daniele A. Molecular Mechanisms Involved in the Positive Effects of Physical Activity on Coping with COVID-19. (2020)

96. Nikhra V. Identifying Patterns in Covid-19: Morbidity, Recovery, and the Aftermath. (2020) doi:10.13140/RG.2.2.30040.93449

97. Nizami NS, Mujeebuddin CS. Strong Immunity - A Major Weapon to Fight against Covid-19. *IOSR Journal Of Pharmacy And Biological Sciences* (2020) **15**:22–29. doi:10.9790/1503032229

98. Pal R, Bhadada SK. Managing common endocrine disorders amid COVID-19 pandemic. *Diabetes & Metabolic Syndrome: Clinical Research & Reviews* (2020) **14**:767–771. doi:10.1016/j.dsx.2020.05.050

99. Palmer K, Monaco A, Kivipelto M, Onder G, Maggi S, Michel J-P, Prieto R, Sykara G, Donde S. The potential long-term impact of the COVID-19 outbreak on patients with non-communicable diseases in Europe: consequences for healthy ageing. *Aging Clin Exp Res* (2020) doi:10.1007/s40520-020-01601-4

100. Parvin F, Islam S, Urmy Z, Ahmed S. The symptoms, contagious process, prevention and post treatment of Covid-19. *European Journal of Physiotherapy and Rehabilitation Studies* (2020) **0**: Available at: https://oapub.org/hlt/index.php/EJPRS/article/view/52 [Accessed May 8, 2020]

101. Pinnock D. Vitamin D and COVID-19. The Evidence Warrants Discussion! *The Medicinal Chef making health simple* (2020) Available at: https://www.themedicinalchef.co.uk/vitamin-d-and-covid-19-the-evidence-warrants-discussion/ [Accessed June 25, 2020]

102. Queiroz NSF, Barros LL, de Azevedo MFC, Oba J, Sobrado CW, de Sousa Carlos A, Milani LR, Sipahi AM, Damião AOMC. Management of inflammatory bowel disease patients in the COVID-19 pandemic era: a Brazilian tertiary referral center guidance. *Clinics (Sao Paulo)* (2020) **75**: doi:10.6061/clinics/2020/e1909

103. Quesada-Gomez JM, Castillo ME, Bouillon R. Vitamin D Receptor stimulation to reduce Acute Respiratory Distress Syndrome (ARDS) in patients with Coronavirus SARS-CoV-2 infections: Revised Ms SBMB 2020_166. *J Steroid Biochem Mol Biol* (2020)105719. doi:10.1016/j.jsbmb.2020.105719

104. Rabbitt L, Slattery E. Vitamin D and Covid-19 – A Note of Caution – Irish Medical Journal. *Irsh Medical Journal* (2020) **113**:82. Available at: http://imj.ie/vitamin-d-and-covid-19-a-note-of-caution/ [Accessed May 14, 2020]

105. Ramos EM, Abreu AC de, Freitas SLF de, Lima MD de, Reis FJM dos, Ramos HV, Souza ID de, Bochenek L de MS, Fonseca AC da, Nascimento VA do. COVID-19, Rate of Case Factors and Nutritional Characteristics of Patients Dying in Italy and Brazil: A Critical Analyze. *Global Journal of Health Science* (2020) **12**:p133. doi:10.5539/gjhs.v12n7p133

106. Raymond M, Ching-A-Sue G, Oliver VH. Mast cell stabilisers, leukotriene antagonists and antihistamines: A rapid review of effectiveness in COVID-19. *Centre for Evidence-Based Medicine* (2020) Available at: https://covid19-evidence.paho.org/handle/20.500.12663/1569 [Accessed June 16, 2020]

107. Razdan K, Singh K, Singh D. Vitamin D Levels and COVID-19 Susceptibility: Is there any Correlation? *Medicine in Drug Discovery* (2020) **7**:100051. doi:10.1016/j.medidd.2020.100051

108. Rhodes JM, Subramanian S, Laird E, Anne Kenny R. Letter: low population mortality from COVID-19 in countries south of latitude 35 degrees North supports vitamin D as a factor determining severity. Authors' reply. *Aliment Pharmacol Ther* (2020) doi:10.1111/apt.15823

109. Ribeiro H, Santana KV de S de, Oliver SL, Rondó PH de C, Mendes MM, Charlton K, Lanham-New S. Does Vitamin D play a role in the management of Covid-19 in Brazil? *Revista de Saúde Pública* **54**: doi:10.11606/s1518-8787.2020054002545

110. Richards G, Mer M, Schleicher G, Stacey S. COVID-19 and the rationale for pharmacotherapy : a South African perspective. *Wits Journal of Clinical Medicine* (2020) **2**:11–18. doi:10.18772/26180197.2020.v2nSIa2

111. Stuart Richer OD. How to build a lifestyle and nutritional firewall against viruses like COVID-19. *Optometry Times* (2020) **12**:1–7. Available at: https://www.optometrytimes.com/article/how-build-lifestyle-and-nutritional-firewall-against-viruses-covid-19 [Accessed June 16, 2020]

112. Rocha JC, Calhau C, MacDonald A. Reply to Jakovac; Severity of COVID-19 infection in patients with phenylketonuria: is vitamin D status protective? *American Journal of Physiology-Endocrinology and Metabolism* (2020) **318**:E890–E891. doi:10.1152/ajpendo.00195.2020

113. Romano L, Bilotta F, Dauri M, Macheda S, Pujia A, De Santis GL, Tarsitano MG, Merra G, Di Renzo L, Esposito E, et al. Short Report - Medical nutrition therapy for critically ill patients with COVID-19. *Eur Rev Med Pharmacol Sci* (2020) **24**:4035–4039. doi:10.26355/eurrev_202004_20874

114. Roy AS, Matson M, Herlekar R. Response to 'Vitamin D concentrations and COVID-19 infection in UK Biobank.' *Diabetes & Metabolic Syndrome: Clinical Research & Reviews* (2020) **14**:777. doi:10.1016/j.dsx.2020.05.049

115. Rusciano D, Bagnoli P, Galeazzi R. The Fight Against COVID-19: The Role of Drugs and Food Supplements. *J Pharmacol Pharm Res* (2020) **3**:5–15. Available at: https://researchopenworld.com/the-fight-against-covid-19-the-role-of-drugs-and-food-supplements/ [Accessed June 17, 2020]

116. Saul AW. Nutritional Treatment of Coronavirus. *Orthomolecular Medicine News Service* (2020) Available at: http://orthomolecular.org/resources/omns/v16n06.shtml

117. Sharma SK, Mudgal SK, Pai VS, Chaturvedi J, Gaur R. Vitamin D: A cheap yet effective bullet against coronavirus disease-19–Are we convinced yet? *National Journal of Physiology, Pharmacy and Pharmacology* (2020) **10**:0–0.

118. Siddiqui R, Ahmed Khan N. Centralized air-conditioning and transmission of novel coronavirus. *Pathog Glob Health* (2020)1–2. doi:10.1080/20477724.2020.1765653

119. Silberstein M. Vitamin D: A simpler alternative to tocilizumab for trial in COVID-19? *Med Hypotheses* (2020) **140**:109767. doi:10.1016/j.mehy.2020.109767

120. Silberstein M. Premorbid IL-6 levels may predict mortality from COVID-19. (2020) doi:10.21203/rs.3.rs-31814/v1

121. Siuka D, Pfeifer M, Pinter B. Vitamin D supplementation in the COVID-19 pandemic. *Mayo Clinic Proceedings* (2020) **0**: doi:10.1016/j.mayocp.2020.05.036

122. Sparavigna, Amelia Carolina. Vitamin D for Covid-19? *Politecnico Di Torino* (2020) doi:10.5281/ZENODO.3822187

123. Speeckaert MM, Delanghe JR. Association between low vitamin D and COVID-19: don't forget the vitamin D binding protein. *Aging Clin Exp Res* (2020) doi:10.1007/s40520-020-01607-y

124. Suresh PS. Hypovitaminosis D and COVID-19: Matter of Concern in India? *Ind J Clin Biochem* (2020) doi:10.1007/s12291-020-00894-6

125. Taheri S, Chagoury O, Tourette M, Skaroni I, Othman M, Bashir M, Dukhan K, Mohannadi DA, Hassan D, Ghadban W, et al. Managing diabetes in Qatar during the COVID-19 pandemic. *The Lancet Diabetes & Endocrinology* (2020) **0**: doi:10.1016/S2213-8587(20)30154-6

126. Tan SHS, Hong CC, Saha S, Murphy D, Hui JH. Medications in COVID-19 patients: summarizing the current literature from an orthopaedic perspective. *Int Orthop* (2020) doi:10.1007/s00264-020-04643-5

127. Thomson B. The COVID-19 Pandemic: A Global Natural Experiment. *Circulation* (2020) doi:10.1161/CIRCULATIONAHA.120.047538

128. Tian Y, Rong L. Letter: Covid-19 and vitamin D-authors' reply. *Aliment Pharmacol Ther* (2020) doi:10.1111/apt.15764

129. Tian Y, Rong L. Letter: does vitamin D have a potential role against COVID-19? Authors' reply. *Aliment Pharmacol Ther* (2020) doi:10.1111/apt.15817

130. Trinity College Dublin. Vitamin D determines severity in COVID-19 so government advice needs to change. *EurekAlert!* Available at: https://www.eurekalert.org/pub_releases/2020-05/tcd-vdd051220.php [Accessed May 14, 2020]

131. Turashvili N, Javashvili L. Could vitamin D reduce the risk of COVID-19? *Translational and Clinical Medicine - Georgian Medical Journal* (2020) **5**:4–6. Available at: http://tcm.tsu.ge/index.php/TCM-GMJ/article/view/204 [Accessed May 8, 2020]

132. Uçar D, Tayfun K, Müslümanoğlu AY, Kalayci MZ. Coronavirus ve Fitoterapi. *Bütünleyici ve Anadolu Tıbbı Dergisi* (2020) **1**:49–57. Available at: https://dergipark.org.tr/en/pub/batd/711108 [Accessed May 20, 2020]

133. Wang L, Wang Y, Ye D, Liu Q. Review of the 2019 novel coronavirus (SARS-CoV-2) based on current evidence. *International Journal of Antimicrobial Agents* (2020)105948. doi:10.1016/j.ijantimicag.2020.105948

134. Weir EK, Thenappan T, Bhargava M, Chen Y. Does vitamin D deficiency increase the severity of COVID-19? *Clinical Medicine* (2020) doi:10.7861/clinmed.2020-0301

135. Wimalawansa SJ. Global epidemic of coronavirus—Covid-19: What can we do to minimize risks. *European Journal of Biomedical and Pharmaceutical Sciences* (2020) **7**:7. Available at: https://www.researchgate.net/profile/Sunil_Wimalawansa/publication/340102912_EJBPS-Prof_WImalawansa-COVID-19-March_2020s/links/5e78eb434585158bd50069f3/EJBPS-Prof-WImalawansa-COVID-19-March-2020s.pdf

136. Yong SJ. Population Studies Confirm Risk Factors for Catching Covid-19. *Medium* (2020) Available at: https://medium.com/microbial-instincts/population-studies-confirm-risk-factors-for-catching-covid-19-853779e7a3c6 [Accessed June 26, 2020]

137. Yong SJ. Vitamin D as an Independent Risk Factor for COVID-19 Death. *Medium* (2020) Available at: https://medium.com/microbial-instincts/lack-of-vitamin-d-as-an-independent-risk-factor-for-covid-19-death-82365d0520fa [Accessed June 28, 2020]

138. Yousfi N, Bragazzi NL, Briki W, Zmijewski P, Chamari K. The COVID-19 pandemic: how to maintain a healthy immune system during the quarantine - a multidisciplinary approach with special focus on athletes. *bs* (2020) doi:10.5114/biolsport.2020.95125

139. Zabetakis I, Lordan R, Norton C, Tsoupras A. COVID-19: The Inflammation Link and the Role of Nutrition in Potential Mitigation. *Nutrients* (2020) **12**: doi:10.3390/nu12051466

140. Zemb P, Bergman P, Camargo CA, Cavalier E, Cormier C, Courbebaisse M, Hollis B, Minisola S, Pilz S, Pludowski P, et al. Vitamin D deficiency and COVID-19 pandemic. *J Glob Antimicrob Resist* (2020) doi:10.1016/j.jgar.2020.05.006

141. Zhang J, Xie B, Hashimoto K. Current status of potential therapeutic candidates for the COVID-19 crisis. *Brain Behav Immun* (2020) doi:10.1016/j.bbi.2020.04.046

142. Zhang L, Liu Y. Potential interventions for novel coronavirus in China: A systematic review. *J Med Virol* (2020) **92**:479–490. doi:10.1002/jmv.25707

143. Ziccarelli vince. Nutrition Therapy for Severe Viral Infections (COVID-19): Recommendations and Considerations for Integrative Medical Treatments. *Journal of Orthomolecular Medicine* (2020) **35**: Available at: https://isom.ca/article/nutrition-therapy-for-severe-viral-infections-covid-19/ [Accessed June 16, 2020]
