# Supplementary material for: A Basic Review of the Preliminary Evidence That COVID-19 Risk and Severity Is Increased in Vitamin D Deficiency
Source: Front Public Health. 2020 Sep 10;8:513. doi: 10.3389/fpubh.2020.00513 (PMC7513835; doi:10.3389/fpubh.2020.00513)
Supplement: Supplementary file 2 [file Table_2.pdf]

**Table A2: Brief Summaries of Original Research (in vitro research is included in Table A1) (47 entries)**					
1st Author /Location	Title (Truncated)	Number of Participants /Type*/ #pages	Supporting Information**	Results/ Recommendation Regarding Covid-19	Support for Supplementation Now
Ahmed/ UK	First Covid-19 maternal mortality …thrombotic complications(1)	1/CS 4 pages	T	Thrombosis kills/ Vigilance is needed	5
Alipio/ Philippines	Vitamin D … possibly improve clinical outcomes of patients infected with … (Covid-2019)(2)	212/R 6 pages	R,C	Sig. D-outcome link /D suppl. could improve outcomes	8
Alipio/ Philippines	Do latitude and ozone concentration predict Covid-2019 cases in 34 countries?(3)	34 countries /A 9 pages	R,	Sig Covid-19-UV (D) link, no latitude link /D suppl. indicated	7
Annweiler/France & China	Point of view: Should COVID-19 patients be supplemented with vitamin D?(4)	6 of 7 causal criteria/C 25 pages	G,P,R,F,C,J,S	Strong biological plausibility case - supplement D	9
Asyary/ Indonesia	Sunlight exposure increased Covid-19 recovery rates: A study in … Indonesia(5)	9 hospitals /R 4 pages	G,I,R,F,	Patient exposure to sunshine helped Encourage it	7
Bäcker/ USA (see note)	Why COVID-19 May Be Disproportionately Killing African Americans…Irradiance…Income(6)	8 locations in the USA/ A 6 pages	G,I	Irradiation for disinfection, sun for protection	6
Bäcker/ USA (see note)	Slower COVID-19 morbidity and mortality growth at higher solar irradiance and elevation(7)	159 locations/A 21 pages	G,I,	Encourage people to go where the sun is overhead	6
ann/ USA (see note)	Non-black Hispanic/Latinos Spend More Time Outdoors … Lower … Morbidity Than Non-Hispanic…(8)	All of Georgia /A 7 pages	G,I,P	Urgently test sunlight exposure, especially for African Americans	6
Bäcker/ USA	Double COVID-19 … Fatality Rate in Countries with High Elderly Female Vitamin D Deficiency Prevalence(9)	32 countries /A 11 pages 1	G,I,P,C,S	Test Vitamin D for prevention and treatment	6

Bossoni/ Italy	Severe hypocalcemia in a thyroidectomized woman with Covid-19 infection(10)	1/CS<br>2 pages	R	Warn thyroid patients due to home confinement	8
Braiman/ USA	Latitude … COVID-19 mortality rate—a possible relationship to vitamin D deficiency?(11)	all nations (WHO data) P/ 14 pages	G	Test for link by supplementing and looking for changes	7
Cuñat/ Spain	Vitamin D deficiency in critically ill patients … COVID -19. Are we doing enough? … 226 patients.(12)	226 patients (subset: 17) /R/7 pages	P,R,C,S	Should assess D more often – all 17 were deficient	8
Daneshkhah/USA	The Possible Role of Vitamin D in Suppressing Cytokine Storm and … Mortality in COVID-19 Patients(13)	10 countries /A/<br>23 pages	C	Study if Vitamin D deficiency speeds mortality	6
Darling /UK	Vitamin D status, body mass index, ethnicity and COVID-19: … UK Biobank COVID-19 …(n 580) … negative controls (n 723)(14)	580 Covid+ vs 580 negative /R 3 pages	None mentioned	All identified risk factors are uncontrollable - No recommendations	0
Davies/UK<br>Switzerland	Evidence Supports a Causal Model for Vitamin D in COVID-19 Outcomes(15) (two studies)	Global data /A and C/ 30 pages	G,P,R	D deficiency is a cause of Covid-19<br>Give up to 4000IU	9
D'Avolio/<br>Switzerland, Italy	25-Hydroxyvitamin D ..Are Lower in Patients with Positive PCR for SARS-CoV-2(16)	107 Covid+ vs 1377 /R/ 7 pages	R	D correlated to + Supplements urged as per Grant, et al.	9
De Smet/ Belgium	Vitamin D deficiency as risk factor for severe 1 COVID-19: a convergence of two pandemics(17)	186 Covid+ vs 2717 /R/ 23 pages	D,P,R,C,	Deficiency seems to cause severe, supplements urged	9
Fasano/<br>Italy,<br>Canada	COVID-19 in Parkinson's Disease Patients Living in … Italy(18)	105 Covid+ vs 1381/S/ 12 pages	G,C	Parkinson's doesn't matter; D does.<br>Conduct D studies	7
Faul/ Ireland	Vitamin D Deficiency and ARDS after SARS-CoV-2 Infection(19)	33 Covid+<br>S/ 2 pages	D,C	Worse outcomes in men with lower D	7

Fox/ USA	No Association …Between Vitamin D Deficiency and COVID-19 Infection, Hospitalization, or Mortality(20)	28,185 patients<br>3 pages	None mentioned	No difference with deficiency, but authors call for better studies	0
Ghasemian/ Iran, Australia, Russia,	The Role of Vitamin D in The Age of COVID-19: A Systematic Review and Meta-Analysis Along with an Ecological Approach(21)	9 studies (subset:6)<br>51 countries<br>20 pages	G,P,R,C,	Patients with Covid-19 lacked D, slight country link<br>Need RCTs	6
Glicio/ India	Vitamin D Level of Mild and Severe Elderly Cases of COVID-19…(22)	176 Covid+<br>18 pages	P,R,C,	Link is suggested<br>Supplement elderly	6
Hastie/<br>UK	Vitamin D concentrations and COVID-19 infection in UK Biobank(23)	449 Covid+ vs 348,149 R/5 pages	G,P,	Link attributed to confounds – D has no role in Covid-19	0
Horowitz/ USA	Efficacy of glutathione ..dyspnea … COVID-19 pneumonia … 2 cases(24)	2 (1 D)<br>8 pages	None mentioned	One patient had D deficiency	5
Ilie/UK	The role of vitamin D in the prevention of coronavirus disease 2019 infection and mortality(25)	20 countries /P/<br>4 pages	D,G,P,R,C,S	Recommend study comparing D levels & Covid-19 severity	6
Kara/Italy,Turkey, Taiwan	'Scientific Strabismus' or Two Related Pandemics COVID-19 & Vitamin D Deficiency(26)	40 countries /P/<br>6 pages	D,G,P,R,S	Goal 40-60ng/ml 10,000IU/day x1mo then 5000IU/day	10
Kohlmeier/ USA	Avoidance of vitamin D deficiency to slow the COVID-19 pandemic(27)	22 states/A/<br>7 pages	D,G,P,R,C	Precision nutrition RDA - <4000IU/Day	8
Kumar/ India	Spurious Correlation? A review of the relationship … Vitamin D and Covid-19 infection & mortality(28)	20 countries /P/<br>7 pages	None mentioned	Ilie is wrong – no role for vitamin D<br>Overdose is likely	0
Laird/ UK, Ireland	Vitamin D and Inflammation: Potential	12 countries /P/	D,P,G,R,C,S	Correlation exists<br>Publicize D	6

	Implications for Severity of Covid-19(29)	7 pages		recommendations	
Lau/USA	Vitamin D insufficiency is prevalent in severe COVID-19(30)	20 Covid+ R/14 pages	G,P,R,C,T	Worse with low D Study D further	6
Li/ China, USA	Identifying novel factors … COVID-19 using …machine learning(31)	A (machine) 32 pages	Not discussed	D is independent risk factor for cases	7
Li/USA	Multivariate Analysis of Factors Affecting COVID-19 …U.S. Counties: …Black Race and Temperature(32)	661 & 217 counties A/ 21 pages	D,P,R,C	D is a likely factor Conducting studies Supplement all	7
Li/USA, China	Sunlight and vitamin D in … (COVID-19) infection and mortality …(33)	49 states A/ 15 pages	D,G,P,R,C,	Appears related Need more studies	6
Marik/ USA	Does vitamin D status impact mortality from SARS-CoV-2 …?(34)	50 states A/ 2 pages	G,P,R,C	Appears related Standard dosages	6
Meltzer/ USA	Association of Vitamin D Deficiency … Treatment with COVID-19 …(35)	499 patients R/ 22 pages	D,G,P,R,C	D decreases risk 4000-5000IU/day	10
Moozhipurath/ Germany	Evidence of Protective Role of Ultraviolet-B (UVB) Radiation in Reducing COVID-19 Deaths(36)	152 affected countries/A/ 42 pages	R,C,F	D decreases deaths Encourage time in sun for deficient	6
Notari/ Spain, Brazil	COVID-19 transmission risk factors(37)	126 countries, 24 factors A/ 42 pages	R	Independent risk: Type 1 diabetes, BCG vaccination, and vitamin D	7
Panarese/ Italy	Letter: Covid-19, and vitamin D (response to Tian, et al. Gastro)(38)	108countries A/3 pages	G,P,R,C,S	Immune control 2000IU/day	8
Pinzon/ Indonesia	Vitamin D Deficiency … Patients with COVID-19 : Case Series and Recent Literature Review(39)	10 patients CS 9 pages	D,G,P,R,C	Immune benefits They give all patients 2000IU	8
Raharusuna/ Indonesia	Patterns of COVID-19 Mortality and Vitamin D: An Indonesian Study(40)	780 patients R 14 pages	R,C	D is strongly associated with death – do RCTs	8

Raisi-Estabragh/ UK	Greater risk of severe COVID-19 in non-White ethnicities is not explained by …vitamin D status: study of 1,326 … UK Biobank(41)	Hospitalized 1326 +<br>3184 -<br>R/ 21 pages	Not discussed	Ruled out cardio-metabolic, social, behavior, and D – leaving genetic?	0
Rhodes/ UK	Editorial: low population mortality …south of latitude 35 degrees North supports vitamin D …(42)	120 countries<br>A/ 4 pages	D,P,C,	Could protect from cytokine storm 1000IU/day	7
Singh/ India	Revisiting the role of vitamin D … prevention and mortality in European …post peak(43)	20 countries<br>2 dates<br>A/ 8 pages	R,C,S	D influenced cases more than deaths study ? of giving D	3
Skutsch/Netherlands, Mexico, Germany	The association of UV with rates of COVID-19 transmission and deaths in Mexico: the possible mediating role of vitamin D(44)	45 cities in Mexico<br>A/<br>29 pages	D,P,R,C,	Further study of link between UV (vitamin D) and transmission rates	6
Sun/ China	Serum calcium as a biomarker of clinical severity and prognosis…(45)	241 patients<br>P/18 pages	not discussed	Deficiency suggests larger studies	7
Tan/<br>Singapore	A cohort study to evaluate the effect of combination Vitamin D, Magnesium and Vitamin B12 (DMB) on progression to severe outcome in older COVID-19 patients(46)	43 patients<br>26 controls<br>17 supplement<br>R/ 6 pages	R,C	Give combination earlier to all cases and to high risk contacts	9
Yao/ China	No Association of COVID-19 transmission with temperature or UV radiation in Chinese cities(47)	224 cities in China/ G/<br>9 pages	G,R	UV (therefore D) not related to transmission	0

*Type of Study
A = Analyses of population data or latitude data (geographic)
C = Causal inference modeling report, Hill's methodology for exploring causality, etc.
P = Prospective correlational study
R = Retrospective chart (or data) review
CS = Single or Multiple Case Study

** Supporting Information provided by these authors:
G = geographical observations (not research) of relationship between low vitamin D and high Covid-19
I = Irradiance in the geographic area influences vitamin D deficiency
Biological plausibility:
R = vitamin D enhances resistance to respiratory viruses, decreasing incidence of infection
F = vitamin D decreases overall fatalities from respiratory viruses
C = vitamin D suppresses cytokines that are implicated in severe Covid-19
J = vitamin D tightens junctions, helping prevent viral infections from progressing to pneumonia
T = vitamin D decreases the risk of thrombosis
S = vitamin D suppresses the 'Renin-Angiotensin' System activity, which is more of a problem for males

Note: Content from an earlier preprint by Bäcker, Follow the Sun: Slower COVID-19 morbidity and mortality growth at higher irradiances, https://ssrn.com/abstract=3567587, last revised 13 April 2020, appears to have been divided into the two much more complete and more recently revised articles included here in Table 3. Similarly, Bäcker's preprint, Non-black Latinos Spend More Time Outdoors and Have Lower COVID-19 Morbidity and Case Fatality Rates Than Non-Latinos, last revised April 16, appears to have been completely incorporated into a similar work, Non-black Hispanic/Latinos Spend More Time Outdoors and Have Lower COVID-19 Morbidity Than Non-Hispanic/Latinos, Last revised April 20, 2020.

References

1. Ahmed I, Azhar A, Eltaweel N, Tan BK. First Covid-19 maternal mortality in the UK associated with thrombotic complications. *Br J Haematol* (2020) doi:10.1111/bjh.16849

2. Alipio M. Vitamin D Supplementation Could Possibly Improve Clinical Outcomes of Patients Infected with Coronavirus-2019 (COVID-2019). Rochester, NY: Social Science Research Network (2020). doi:10.2139/ssrn.3571484

3. Alipio MM. Do latitude and ozone concentration predict Covid-2019 cases in 34 countries? *medRxiv* (2020)2020.04.09.20060202. doi:10.1101/2020.04.09.20060202

4. Annweiler C, Cao Z, Sabatier J-M. Point of view: Should COVID-19 patients be supplemented with vitamin D? *Maturitas* (2020) **140**:24–26. doi:10.1016/j.maturitas.2020.06.003

5. Asyary A, Veruswati M. Sunlight exposure increased Covid-19 recovery rates: A study in the central pandemic area of Indonesia. *Sci Total Environ* (2020) doi:10.1016/j.scitotenv.2020.139016

6. Bäcker A. Why COVID-19 May Be Disproportionately Killing African Americans: Black Overrepresentation among COVID-19 Mortality Increases with Lower Irradiance, Where Ethnicity Is More Predictive of COVID-19 Infection and Mortality Than Median Income. Social Science Research Network (2020). doi:10.2139/ssrn.3571699

7. Bäcker A. Slower COVID-19 Morbidity and Mortality Growth at Higher Solar Irradiance and Elevation. Rochester, NY: Social Science Research Network (2020). doi:10.2139/ssrn.3604729

8. Bäcker A. Non-black Hispanic/Latinos Spend More Time Outdoors and Have Lower COVID-19 Morbidity Than Non-Hispanic/Latinos. Rochester, NY: Social Science Research Network (2020). doi:10.2139/ssrn.3575217

9. Bäcker A, Mageswaran M. Double COVID-19 Confirmed Case Fatality Rate in Countries with High Elderly Female Vitamin D Deficiency Prevalence. Rochester, NY: Social Science Research Network (2020). doi:10.2139/ssrn.3623662

10. Bossoni S, Chiesa L, Giustina A. Severe hypocalcemia in a thyroidectomized woman with Covid-19 infection. *Endocrine* (2020)1–2. doi:10.1007/s12020-020-02326-0

11. Braiman M. Latitude Dependence of the COVID-19 Mortality Rate—A Possible Relationship to Vitamin D Deficiency? Rochester, NY: Social Science Research Network (2020). doi:10.2139/ssrn.3561958

12. Cuñat T, Ojeda A, Calvo A. Vitamin D deficiency in critically ill patients diagnosed with COVID -19. Are we doing enough? A retrospective analysis of 226 patients. In Review (2020). doi:10.21203/rs.3.rs-30390/v1

13. Daneshkhah A, Agrawal V, Eshein A, Subramanian H, Roy HK, Backman V. The Possible Role of Vitamin D in Suppressing Cytokine Storm and Associated Mortality in COVID-19 Patients. *medRxiv* (2020)2020.04.08.20058578. doi:10.1101/2020.04.08.20058578

14. Darling AL, Ahmadi KR, Ward KA, Harvey NC, Alves AC, Dunn-Waters DK, Lanham-New SA, Cooper C, Blackbourn DJ. Vitamin D status, body mass index, ethnicity and COVID-19: Initial analysis of the first-reported UK Biobank COVID-19 positive cases (n 580) compared with negative controls (n 723). *medRxiv* (2020)2020.04.29.20084277. doi:10.1101/2020.04.29.20084277

15. Davies G, Garami AR, Byers JC. Evidence Supports a Causal Model for Vitamin D in COVID-19 Outcomes. *medRxiv* (2020)2020.05.01.20087965. doi:10.1101/2020.05.01.20087965

16. D'Avolio A, Avataneo V, Manca A, Cusato J, De Nicolò A, Lucchini R, Keller F, Cantù M. 25-Hydroxyvitamin D Concentrations Are Lower in Patients with Positive PCR for SARS-CoV-2. *Nutrients* (2020) **12**: doi:10.3390/nu12051359

17. De Smet D, De Smet K, Herroelen P, Gryspeerdt S, Martens GA. Vitamin D deficiency as risk factor for severe COVID-19: a convergence of two pandemics. *medRxiv* (2020)2020.05.01.20079376. doi:10.1101/2020.05.01.20079376

18. Fasano A, Cereda E, Barichella M, Cassani E, Ferri V, Zecchinelli AL, Pezzoli G. COVID-19 in Parkinson's Disease Patients Living in Lombardy, Italy. *Mov Disord* (2020) doi:10.1002/mds.28176

19. Faul JL, Kerley C, Love B, O-Nell E, Cody C, Tormey W, Hutchinson K, Cormican LJ, Burke CM. Vitamin D Deficiency and ARDS after SARS-CoV-2 Infection – Irish Medical Journal. *Irish Medical Journal* (2020) **113**:84. Available at: http://imj.ie/vitamin-d-deficiency-and-ards-after-sars-cov-2-infection/ [Accessed May 14, 2020]

20. Fox B, Sizemore JO. No Association Seen Between Vitamin D Deficiency and COVID-19 Infection, Hospitalization, or Mortality.

21. Ghasemian R, Shamshirian A, Heydari K, Malekan M, Alizadeh-Navaei R, Ebrahimzadeh MA, Jafarpour H, Shahmirzadi AR, Khodabandeh M, Seyfari B. The Role of Vitamin D in The Age of COVID-19: A Systematic Review and Meta-Analysis Along with an Ecological Approach. *medRxiv* (2020)

22. Glicio EJ. Vitamin D Level of Mild and Severe Elderly Cases of COVID-19: A Preliminary Report. Rochester, NY: Social Science Research Network (2020). Available at: https://papers.ssrn.com/abstract=3593258 [Accessed May 20, 2020]

23. Hastie CE, Mackay DF, Ho F, Celis-Morales CA, Katikireddi SV, Niedzwiedz CL, Jani BD, Welsh P, Mair FS, Gray SR, et al. Vitamin D concentrations and COVID-19 infection in UK Biobank. *Diabetes & Metabolic Syndrome: Clinical Research & Reviews* (2020) **14**:561–565. doi:10.1016/j.dsx.2020.04.050

24. Horowitz RI, Freeman PR, Bruzzese J. Efficacy of glutathione therapy in relieving dyspnea associated with COVID-19 pneumonia: A report of 2 cases. *Respiratory Medicine Case Reports* (2020) **30**:101063. doi:10.1016/j.rmcr.2020.101063

25. Ilie PC, Stefanescu S, Smith L. The role of Vitamin D in the prevention of Coronavirus Disease 2019 infection and mortality. In Review (2020). doi:10.21203/rs.3.rs-21211/v1

26. Kara M, Ekiz T, Ricci V, Kara Ö, Chang K-V, Özçakar L. “Scientific Strabismus” or Two Related Pandemics: COVID-19 & Vitamin D Deficiency. *Br J Nutr* (2020)1–20. doi:10.1017/S0007114520001749

27. Kohlmeier M. Avoidance of vitamin D deficiency to slow the COVID-19 pandemic. *BMJ Nutrition, Prevention & Health* (2020)bmjnph-2020-000096. doi:10.1136/bmjnph-2020-000096

28. Kumar V, Srivastava A. Spurious Correlation? A review of the relationship between Vitamin D and Covid-19 infection and mortality. *medRxiv* (2020)2020.05.25.20110338. doi:10.1101/2020.05.25.20110338

29. Laird E, Rhodes J, Kenny RA. Vitamin D and Inflammation – Potential Implications for Severity of Covid-19 – Irish Medical Journal. *Irish Medical Journal* (2020) **113**:81. Available at: http://imj.ie/vitamin-d-and-inflammation-potential-implications-for-severity-of-covid-19/ [Accessed May 14, 2020]

30. Lau FH, Majumder R, Torabi R, Saeg F, Hoffman R, Cirillo JD, Greiffenstein P. Vitamin D Insufficiency is Prevalent in Severe COVID-19. *medRxiv* (2020)2020.04.24.20075838. doi:10.1101/2020.04.24.20075838

31. Li M, Zhang Z, Cao W, Liu Y, Du B, Chen C, Liu Q, Uddin MN, Jiang S, Chen C, et al. Identifying novel factors associated with COVID-19 transmission and fatality using the machine learning approach. *medRxiv* (2020)2020.06.10.20127472. doi:10.1101/2020.06.10.20127472

32. Li AY, Hannah TC, Durbin J, Dreher N, McAuley FM, Marayati NF, Spiera Z, Ali M, Gometz A, Kostman JT, et al. Multivariate Analysis of Factors Affecting COVID-19 Case and Death Rate in U.S. Counties: The Significant Effects of Black Race and Temperature. *medRxiv* (2020)2020.04.17.20069708. doi:10.1101/2020.04.17.20069708

33. Li Y, Li Q, Zhang N, Liu Z. Sunlight and vitamin D in the prevention of coronavirus disease (COVID-19) infection and mortality in the United States. (2020)

34. Marik PE, Kory P, Varon J. Does vitamin D status impact mortality from SARS-CoV-2 infection? *Med Drug Discov* (2020)100041. doi:10.1016/j.medidd.2020.100041

35. Meltzer DO, Best TJ, Zhang H, Vokes T, Arora V, Solway J. Association of Vitamin D Deficiency and Treatment with COVID-19 Incidence. *medRxiv* (2020)2020.05.08.20095893. doi:10.1101/2020.05.08.20095893

36. Moozhipurath RK, Kraft L, Skiera B. Evidence of Protective Role of Ultraviolet-B (UVB) Radiation in Reducing COVID-19 Deaths. *medRxiv* (2020)2020.05.06.20093419. doi:10.1101/2020.05.06.20093419

37. Notari A, Torrieri G. COVID-19 transmission risk factors. *arXiv:200503651 [physics, q-bio, stat]* (2020) Available at: http://arxiv.org/abs/2005.03651 [Accessed May 20, 2020]

38. Panarese A, Shahini E. Letter: Covid-19, and vitamin D. *Aliment Pharmacol Ther* (2020) doi:10.1111/apt.15752

39. Pinzon RT, Angela A, Pradana AW. Vitamin D Deficiency Among Patients with COVID-19 : Case Series and Recent Literature Review. In Review (2020). doi:10.21203/rs.3.rs-29473/v1

40. Raharusun P, Priambada S, Budiarti C, Agung E, Budi C. Patterns of COVID-19 Mortality and Vitamin D: An Indonesian Study. Rochester, NY: Social Science Research Network (2020). doi:10.2139/ssrn.3585561

41. Raisi-Estabragh Z, McCracken C, Bethell MS, Cooper J, Cooper C, Caulfield MJ, Munroe PB, Harvey NC, Petersen SE. Greater risk of severe COVID-19 in non-White ethnicities is not explained by cardiometabolic, socioeconomic, or behavioural factors, or by 25(OH)-vitamin D status: study of 1,326 cases from the UK Biobank. *medRxiv* (2020)2020.06.01.20118943. doi:10.1101/2020.06.01.20118943

42. Rhodes JM, Subramanian S, Laird E, Kenny RA. Editorial: low population mortality from COVID-19 in countries south of latitude 35 degrees North – supports vitamin D as a factor determining severity. *Alimentary Pharmacology & Therapeutics* **n/a**: doi:10.1111/apt.15777

43. Singh S, Kaur R, Singh RK. Revisiting the role of vitamin D levels in the prevention of COVID-19 infection and mortality in European countries post infections peak. (2020) doi:10.21203/rs.3.rs-32484/v1

44. Skutsch M, Dobler C, McCall MBB, Ghilardi A, Salinas-Melgoza MA, McCall MK, Fenner-Sanchez G. The association of UV with rates of COVID-19 transmission and deaths in Mexico: the possible mediating role of vitamin D. *medRxiv* (2020)2020.05.25.20112805. doi:10.1101/2020.05.25.20112805

45. Sun J-K, Zhang W-H, Zou L, Liu Y, Li J-J, Kan X-H, Dai L, Shi Q-K, Yuan S-T, Yu W-K, et al. Serum calcium as a biomarker of clinical severity and prognosis in patients with coronavirus disease 2019: a retrospective cross-sectional study. In Review (2020). doi:10.21203/rs.3.rs-17575/v1

46. Tan CW, Ho LP, Kalimuddin S, Cherng BPZ, Teh YE, Thien SY, Wong HM, Tern PJW, Chay JWM, Nagarajan C, et al. A cohort study to evaluate the effect of combination Vitamin D, Magnesium and Vitamin B12 (DMB) on progression to severe outcome in older COVID-19 patients. *medRxiv* (2020)2020.06.01.20112334. doi:10.1101/2020.06.01.20112334

47. Yao Y, Pan J, Liu Z, Meng X, Wang W, Kan H, Wang W. No Association of COVID-19 transmission with temperature or UV radiation in Chinese cities. *Eur Respir J* (2020) doi:10.1183/13993003.00517-2020
