# Supplementary material for: A Basic Review of the Preliminary Evidence That COVID-19 Risk and Severity Is Increased in Vitamin D Deficiency
Source: Front Public Health. 2020 Sep 10;8:513. doi: 10.3389/fpubh.2020.00513 (PMC7513835; doi:10.3389/fpubh.2020.00513)
Supplement: Supplementary file 3 [file Data_Sheet_1.pdf]

Appendix: The Irish Medical Journal Debate

(Appendix to: A Basic Review of the Preliminary Evidence that COVID-19 Risk and Severity is Increased in Vitamin D Deficiency

McKenna and Flynn began the debate by objecting to McCartney et al.'s recommendation of 800IU-2000IU/day of vitamin D, stating that there is no randomized controlled trial (RCT) evidence to support such high doses, which can be harmful, particularly in children.(1,2) They cited early evidence from TILDA suggesting that 800IU/day is adequate for frail elderly and healthy elderly require only 400IU/day, and that only in the winter or when "cocooning."(1) They also cited Molloy, et al., in asserting that infants should not be given more than 200IU/day, expressing concern that such high doses will result in hypercalcemia in children, falls and fractures in the elderly, and the public may conclude that supplementation with 2000IU/day long-term is safe.(1) McKenna and Flynn advised against sunlight exposure due to skin cancer risks, and concluded by asserting that most adults obtain all the vitamin D they need (with 10ng/ml as their target serum level) from fortified foods, even with "cocooning," but conceded that supplementation with 200-800IU/day would be acceptable for those not eating fortified foods.(1) The authors provided a chart to guide dosages for three groups of adults.(1) McKenna and Flynn did not, however, dispute a link between vitamin D and COVID-19.(1)

McCartney and Byrne responded to McKenna and Flynn, pointing out that they submitted an early draft to the Irish Department of Health prior to publication, and the article was written to guide health care professionals, not the general public.(3) Also, the TILDA researchers now recommend 800 – 1000IU of vitamin D per day for adults over aged 70, and nursing home residents, who are the most frail of all (with a 50% COVID-19 case fatality rate in Ireland), were the target group of their article.(3) Supplementation for children was not discussed in the article.(3,4) They contended that concerns for vitamin D toxicity were overstated based upon the evidence, and pointed out that the 25(OH)D (serum vitamin D) levels required to maintain bone health are far lower than the amounts that have been shown to decrease risk of viral respiratory infection.(3) They concluded by citing a publication, "from one of the world's foremost authorities" recommending 2000IU/day for optimal protection against viral infection.(3)

Next, Laird, et al., presented a graph illustrating the correlation ($p = 0.046$) between low population mean 25(OH)D levels and higher COVID-19 death rates (study details in Section 3.4.1).(5) They detailed several biological plausibility arguments for vitamin D's role in COVID-19 outcomes, emphasizing limiting the potentially damaging cytokine response.(5) They found that the risk groups for severe COVID-19 mirror those for vitamin D deficiency.(5) They concluded by stating that RCTs of vitamin D supplementation would be difficult to conduct during the COVID-19 pandemic, because few appropriately informed people would agree to risk getting the placebo.(5)

Rabbitt and Slattery replied to McCartney, et al.'s April article, stating succinctly that immunonutrition is a field fraught with failures, and other vitamin supplements have proven to be harmful in large doses.(6) They agree that vitamin D is safe, but then describe calls for supplementation to help protect the general population from severe COVID-19 as "folly" due to lack of "clear evidence," referring to Donald Trump.(6)

McCartney and Byrne replied to Rabbitt and Slattery, stating they fail to acknowledge the biological plausibility of vitamin D for COVID-19, which is supported by a very strong body of evidence.(7) They provided summaries of three additional studies, and pointed out that Rabbitt and Slattery conceded that vitamin D is safe; the fact that other choices are not safe is irrelevant.(7)

Faul, et al., concluded the debate with a brief letter supporting the April McCartney article.(8) The strong correlation between the risk factors for increased severity of COVID-19 and vitamin D deficiency led them to prospectively evaluate vitamin D levels in 33 hospitalized COVID-19 pneumonia patients who were all in respiratory failure (study details in section 3.4.4).(8)

1. McKenna MJ, Flynn MAT. Covid-19 Cocooning and Vitamin D Intake Requirements – Irish Medical Journal. *Irish Medical Journal* (2020) **113**:79. Available at: http://imj.ie/covid-19-cocooning-and-vitamin-d-intake-requirements/ [Accessed May 14, 2020]

2. McCartney DM, Byrne DG. Optimisation of Vitamin D Status for Enhanced Immuno-protection Against Covid-19. *Ir Med J* (2020) **113**:58.

3. McCartney DM, Byrne DG. McCartney et al comment on ‘Covid-19, Cocooning and Vitamin D Requirements’ response report – Irish Medical Journal. *Irish Medical Journal* (2020) **113**:80. Available at: http://imj.ie/mccartney-et-al-comment-on-covid-19-cocooning-and-vitamin-d-requirements-response-report/ [Accessed May 14, 2020]

4. Laird E, Kenny RA. Vitamin D deficiency in Ireland – implications for COVID-19. Results from the Irish Longitudinal Study on Ageing (TILDA). The Irish Longitudinal Study on Ageing (2020). doi:10.38018/TildaRe.2020-05

5. Laird E, Rhodes J, Kenny RA. Vitamin D and Inflammation – Potential Implications for Severity of Covid-19 – Irish Medical Journal. *Irish Medical Journal* (2020) **113**:81. Available at: http://imj.ie/vitamin-d-and-inflammation-potential-implications-for-severity-of-covid-19/ [Accessed May 14, 2020]

6. Rabbitt L, Slattery E. Vitamin D and Covid-19 – A Note of Caution – Irish Medical Journal. *Irsh Medical Journal* (2020) **113**:82. Available at: http://imj.ie/vitamin-d-and-covid-19-a-note-of-caution/ [Accessed May 14, 2020]

7. McCartney DM, Byrne DG. McCartney et al comment on ‘Vitamin D and Covid-19 – A Note of Caution’ response letter – Irish Medical Journal. *Irish Medical Journal* **113**:79. Available at: http://imj.ie/mccartney-et-al-comment-on-vitamin-d-and-covid-19-a-note-of-caution-response-letter/ [Accessed May 14, 2020]

8. Faul JL, Kerley C, Love B, O-Nell E, Cody C, Tormey W, Hutchinson K, Cormican LJ, Burke CM. Vitamin D Deficiency and ARDS after SARS-CoV-2 Infection – Irish Medical Journal. *Irish Medical Journal* (2020) **113**:84. Available at: http://imj.ie/vitamin-d-deficiency-and-ards-after-sars-cov-2-infection/ [Accessed May 14, 2020]
